# NK cell hyperactivation drives macrophage repolarization and limits M2 bias in pemphigus vulgaris

**DOI:** 10.3389/fimmu.2026.1774059

**Published:** 2026-05-08

**Authors:** Vishakha Hooda, Aishwarya Singh, Sujay Khandpur, Sudheer Arava, Taruna Madan, Alpana Sharma

**Affiliations:** 1 Department of Biochemistry, All India Institute of Medical Sciences, New Delhi, India; 2 Department of Dermatology and Venereology, All India Institute of Medical Sciences, New Delhi, India; 3 Department of Pathology, All India Institute of Medical Sciences, New Delhi, India; 4 Division of Development Research, Indian Council of Medical Research, New Delhi, India

**Keywords:** autoimmunity, macrophages NK crosstalk, monocytes, natural killer cells, pemphigus vulgaris

## Abstract

**Background:**

Macrophages and natural killer (NK) cells are innate immune components proven to be either pathogenic, protective, or both in autoimmune disorders. Nonetheless, the function of macrophages in the pathogenesis of pemphigus vulgaris (PV), an autoimmune skin disorder, is lacking.

**Objectives:**

We aimed to investigate macrophages and their crosstalk with NK cells in PV, with a particular focus on how these interactions influence their functionalities.

**Methods:**

Flow cytometry and immunofluorescence was utilised to assess macrophage profile along with expression of receptors on NK cells and macrophages. *In-vitro* macrophage skewing, and NK–macrophage interactions (including F-actin accumulation, activation markers, cytotoxicity, IFNγ and perforin secretion, *NOS2, CD86, CD206* expression, phagocytic activity, and arginase activity were examined. NKG2D blocking and related assays were performed to elucidate its role in crosstalk.

**Results:**

Expression of NKG2D on NK cells and MICA/B on macrophages was upregulated. CD56^+^ NK cells and CD68^+^ macrophages co-localized in lesions. A significant increase in macrophages in both the periphery and lesional skin was observed. Both M1 and M2 macrophages were upregulated in blood, whereas M1 increased and M2 decreased in skin lesions. Macrophages displayed skewing toward an M2 phenotype *in vitro*. Upon co-culturing, F-actin accumulation increased during interaction. The interaction led to increased activation, cytotoxicity, and IFNγ and perforin secretion in NK cells when M1 stimulation and Dsg3 autoantigen were given. In macrophages, increased expression of iNOS and CD86 and higher phagocytic activity were noted, whereas arginase activity and expression along with CD206 were downregulated. NKG2D blockade on NK cells resulted in partial but significant decrease in NK cells activity including activation, perforin, cytotoxicity and IFNγ release. The reduction were prominent in M1 state. M1 like markers *NOS2* and *CD86* were reduced and arginase activity non significantly improved under M2 state post blockade.

**Conclusion:**

This study helped in comprehending functionally active NK- macrophage axis, where NK cells promote pro inflammatory M1 state, and dysregulate M2 macrophage functionality. NKG2D blockade resulted in partial reversal suggesting its critical, though not elusive, contribution indicating multifactorial crosstalk mechanism. Targeting NK cell activity or promoting M2 functionality could potentially lead to improvements in disease pathogenesis.

## 1 Introduction

Desmoglein are the cell adhesion desmosomal proteins responsible for maintaining the integrity of skin and mucosal membranes (1). In an autoimmune condition, Pemphigus vulgaris (PV), this integrity gets broken by the formation of autoantibodies, particularly IgG4, against desmoglein (Dsg) 1 and Dsg3, causing acantholysis (2). PV is a rare and chronic autoimmune blistering condition encompassing blister or erosion formation on mucosa and skin with a characteristic Nikolky’s sign (3). Approximately 50 to 70 percent of cases show first manifestation on oral mucosa which could sustain for months before showing signs on skin or other mucosal membranes like pharynx, conjunctiva, esophagus, larynx, anus etc (4, 5). In absence of adequate treatment, it can be fatal due to involvement of excessive skin area constituting loss of epidermal barrier which increases susceptibility to secondary infections and most commonly bacterial infestation which may lead to sepsis (4). PV remains a complex disorder in terms of immune response generated. Lack of knowledge of the immune components involved and variations in patient cohorts pose a challenge to develop a potential therapeutic. Consequently, our laboratory has been working on this disease for years and was able to identify significance of immune cells like natural killer (NK) cells (6, 7), innate lymphoid cells (ILCs) (8), dendritic cells (9), gamma delta T cells (10, 11), Th1/Th2 (12), Th17 and Treg cells in PV immunopathogenesis (13–15).

However, the importance of macrophages remains neglected in PV, with only a few studies noting their presence and none elucidating their functional significance (16–18). Monocytes or macrophages in peripheral circulation can be commonly characterized by CD14, a glycoprotein receptor which recognizes pathogen/damage associated molecular patterns causing activation of innate immunity and CD11b, protein responsible for migration, phagocytosis and immune response (19–23). Macrophages have been extensively studied in autoimmune diseases such as systemic sclerosis (24–26), rheumatoid arthritis (27–30), type 1 diabetes (31, 32), and systemic lupus erythematosus (33, 34). At the onset of these conditions, M1 macrophages, known for their pro-inflammatory functions, secrete a wide array of chemokines that recruit other immune cells—such as T cells, B cells, neutrophils, NK cells, and NKT cells—to the site of inflammation. They further activate these immune cells by releasing pro-inflammatory cytokines including IL-1β, IL-6, IL-12, IL-23, IFN-γ, and TNF-α, and through direct interactions involving antigen presentation (via MHC), co-stimulatory molecules (CD80, CD86, CD40). Additionally, M1 macrophages contribute to tissue injury by producing matrix metalloproteinases (MMPs) and reactive oxygen species (ROS). This concerted activation of macrophages and immune cells exacerbates tissue damage. In contrast, M2 macrophages are involved in immunosuppression and tissue repair, primarily by secreting anti-inflammatory cytokines like IL-10 and TGF-β and growth factors such as PDGF and VEGF. M2 macrophages also produce pro-fibrotic mediators, including TGF-β, PDGF, and VEGF, which activate myofibroblasts, promote extracellular matrix deposition, and drive tissue fibrosis (35).

Moreover, crosstalk studies involving interactions between cells gives a real time image and simplifies the mechanisms involved in the pathogenesis of complex disorders helping in development of novel therapeutics. In human body, numerous cells operate based on their environmental context, and their function is influenced by interactions that can happen either through cell-cell contact, involving diverse ligands and receptors, or via soluble mediators such as cytokines (36). The crosstalk between any two immune cells affects not only their own function and signalling but also the behaviour of other cells.

Although the interaction between NK cells and macrophages is not typical, it plays a crucial role in the development of diseases. Previous reports on microbial infections have demonstrated macrophages release cytokine stimulators such as IL12, IL15 and IL18 and engage in receptor- ligand interaction using surface markers like CD48, CD40, MICA/B with their counterparts such as 2B4, CD154, NKG2D on NK cells, resulted in release of cytokines such as IFNγ and enhanced degranulation from NK cells, which in turn could enhance inflammatory pathway and M1 macrophages activity (36–38). Contrastingly, tumor associated macrophages particularly M2 phenotype tend to suppress NK cells cytotoxicity and function (39, 40). Moreover, reports have also suggested activation of NK cells against tumor via macrophages interaction (36, 41, 42). However, their crosstalk in autoimmune diseases remain underexplored. As key components of the innate immune system, these cells can work together to identify and eradicate pathogens, forming an essential part of the body’s initial defense mechanism. Macrophages neutralize the pathogen by phagocytosis while NK cells can directly eliminate the pathogen by their cytolytic properties (38). These cells can work in coordination or opposition thereby affecting each other’s functionalities. These innate cells can interact via cell to cell contact and further secrete certain chemokines and cytokines to bring other immune components to the site of infection. Secretion of a mixture of pro inflammatory and anti-inflammatory soluble mediators in coordination by these innate sentinels modulate the immune responses and determine the characteristics of the adaptive immune system then generated (43, 44).

The phenotypic characteristics and functional role of NK cells in PV has already been studied by our group. We observed an increase in CD56+ CD3- NK cells and their subtypes. In NK cell culture supernatants, IFNγ and perforin levels were significantly increased in PV. NK cells displayed higher activation and cytotoxicity in PV, resulting in promotion of disease pathogenesis (6). Nonetheless, till date, no study has directly correlated the implication of macrophages and its interplay with NK cells in the context of PV. The lack of immunological role and influence of macrophages and their interaction with NK cells encouraged us to investigate their role in an autoimmune disease - PV. This study aims to explore the phenotypic and functional characteristics of macrophages along with their interplay with NK cells. Therefore, studying these interactions in PV will greatly help in providing a deeper understanding of the disease pathogenesis which can help in finding the possible novel therapeutic targets.

## 2 Methods

### 2.1 Study subjects

This study recruited 35 PV patients ranged from 23 to 65 years of age, with a median age of 42 years receiving treatment at the Department of Dermatology and Venereology, All India Institute of Medical Sciences, New Delhi (**Supplementary Table 2**). The institutional ethical committee approved the study (IECPG-144/23.04.2020, OT-14/23.06.2021), and all subjects provided informed written consent. Only patients with active PV, clinically diagnosed and histologically confirmed by hematoxylin and eosin (H&E) staining and direct immunofluorescence (DIF) were included. Additionally, desmoglein 3 antibodies levels were detected (**Supplementary Figure 5**). These patients had not received any immunosuppressive medications for the past 1–3 months and did not have any other inflammatory or autoimmune conditions. They were either treatment naïve or receiving systemic steroids at a dose less than 30 mg/day. They had not been previously included in any published reports. Additionally, 35 healthy volunteers without known diseases were recruited as healthy controls. Blood samples of 10 ml were collected from each subject for routine flow cytometry and culture experiments, with 8 ml in sterile EDTA vials for peripheral blood mononuclear cell (PBMC) isolation and 2 ml for serum isolation. However, for the co-culture experiments of functional assays approximately 20-25ml blood was collected. Additionally, 4 mm biopsies were collected.

### 2.2 Phenotypic characterization using flow cytometry

A combination of antibodies from Invitrogen was used to detect macrophages like monocytes in blood (**Supplementary Table 3**). Previously reported protocol and antibody panel was used (22). Anti-CD11b- BV421, anti-CD14- Super Bright 780, anti HLA DR- APC-eflour 780, anti-CD64- PE Texas red/FITC, anti-CD86- Super Bright 645, anti-CD163- AlexaFlour700 and anti-CD206-PECy7. To study the receptor ligand levels, anti-NKG2D- BV 421, anti-KIR2D- PerCPCy5.5 and anti-MICA/B- PE were used. NK cells activation status was checked using anti-CD69 tagged with PE Cy7. PBMCs were isolated from the whole blood collected in EDTA vial using ficoll gradient method. Primary culture cells were also harvested post culture and centrifuged with 1xPBS buffer at 1500rpm for 5 minutes at room temperature (RT). Prior to staining, a viability dye (Live/Dead™ fixable blue dead cell stain) was added to the PBMCs/Primary culture cells and incubated for 30 minutes, followed by addition of 500ul FACS buffer and centrifugation at 1500 rpm for 5 minutes at 4 °C. Supernatant was discarded and the cells were then incubated with the antibodies in the dark at 4 °C for 45 minutes. After incubation, 500 ul FACS buffer was added to the sample vial and centrifuged for 5 minutes at 4 °C followed by resuspension of the cell pellet in 500ul FACS buffer for acquisition on the BD LSR Fortessa x20 flow cytometer. A total of 1 x 10^5^ events per sample were acquired. The gating strategy has been shown in **Supplementary Figure 1** along with fluorescence minus one in **Supplementary Figure 2**. The acquired data was subsequently analyzed using FlowJo software 10.8 software.

### 2.3 Primary cell culture for NK cells and macrophages

For this study, NK cells were isolated using the EasySep™ Human NK Cell Isolation Kit (Stemcell Technologies, Canada) following the manufacturer’s protocol (**Supplementary Figure 3**). The NK cells were derived from PBMCs through MACS sorting and then cultured in RPMI 1640 media (Himedia). The purified NK cells (1x10^6^ cells/ml) were maintained in complete RPMI media supplemented with IL-2 (100 U/ml), IL-15 (10 ng/ml), and 2% autologous serum. The culture period lasted for 12–14 days, with media changes every 2 days. After this period, the cells were harvested and centrifuged with 1xPBS for downstream applications.

Macrophages were obtained from monocytes present in peripheral circulation. They were cultured in RPMI 1640 media (#AL162S Himedia) containing L-glutamine, 4.5gms per litre glucose, 10mM HEPES, 1.5gms per litre sodium bicarbonate and sodium pyruvate. Firstly, PBMCs were isolated and plated in 12-well plates (approximately 2 x 10^5^ cells per well) and incubated for at least 1 hour to allow monocytes present in PBMCs to adhere to cell culture plate in an incubator supplied with 5% CO_2_ at 37 °C. Subsequently after an hour, non-adherent cells were pipetted out, followed by a gentle wash with 1x PBS using swirling movement and then adding fresh media containing RPMI 1640, 2% human autologous serum, 1% penicillin-streptomycin, and 25 ng/ml M-CSF, followed by incubation. The media was changed every 2 days. By day 8, monocyte derived macrophages were obtained which were either harvested for real time PCR or/and further polarized for functional assays. Macrophages were polarized towards the M1 phenotype using 10 ng/ml LPS and 20 ng/ml IFNγ, or towards the M2 phenotype using 20 ng/ml IL-4 and 10 ng/ml IL-10 in presence or absence of desmoglein 3 antigen. Polarised macrophages were obtained at day 9 and subsequently used for downstream functional assays.

### 2.4 Blocking of NKG2D on NK cells

The sorted NK cells (1x10^6^ cells/ml) were cultured in RPMI media containing 10ug/ml anti NKG2D antibody (catalogue no. 14-5878-82, Invitrogen) for 24 hours at 37 °C in the incubator supplemented with 5% CO2. The cells were harvested for flow cytometry and co-culture functional assays.

### 2.5 Co-culture of NK cells and macrophages

For assessing the impact of crosstalk on NK and macrophages, samples were collected from the patients who were severely to extensively affected by PV, scored by the PDAI. After culturing NK cells and generating macrophages from monocytes for 9–10 days, NK cells were counted and plated with Macrophages in 1:1 ratio for 24 hours (**Supplementary Figure 4**) with the following stimulations: Condition 1 – without co-culture (Only NK/Only MO), Condition 2- without stimulation co-culture (NK+ MO), Condition 3- M1 macrophages polarized with 10ng/ml IFNγ and 20ng/ml LPS co-culture (NK+M1), Condition 4- M1 macrophages polarized with 10ng/ml IFNγ and 20ng/ml LPS and co-culture stimulated with 10ng/ml Dsg3 (NK+ M1+ Dsg3), Condition 5- M2 macrophages polarized with 10ng/ml IL 4 and 20ng/ml IL 10 co-culture (NK + M2) and Condition 6- M2 macrophages polarized with 10ng/ml IL 4 and 20ng/ml IL 10 and co-culture stimulated with 10ng/ml Dsg3 (NK+ M2+ Dsg3). After 24 hours, NK cells were separated from Macrophages by adding 1x PBS and incubated for 30 mins at 4 degrees. Free floating NK cells were then harvested; macrophages adhered to culture plate were gently scraped off and harvested separately. Both cell types were separately centrifuged at 1500rpm in PBS before use.

### 2.6 Quantification of macrophage associated differentially expressed genes

RNA was extracted from the blood and tissue biopsies using Trizol method. Subsequently, the extracted RNA underwent reverse transcription to generate complementary DNA (cDNA) employing MuLV Reverse Transcriptase (Fermentas, USA). The cDNA served as a template for real-time PCR (BioRad, USA) utilizing primers specific to various molecules (**Supplementary Table 1**). Maxima SYBR Green qPCR Master Mix (2X) from Fermentas, USA, facilitated the relative expression analysis of macrophage-associated molecules. The resulting data were normalized to the housekeeping gene 18S and presented as 2-ΔCt values.

### 2.7 Enzyme linked immunosorbent assay (ELISA) to detect cytokine levels

Commercially available high sensitivity ELISA kits from ELabsciences and ThermoFisher were used to detect levels of cytokines secreted in the culture supernatants. ELISA assays were conducted for IFNγ, Perforin, TNFα, TGFβ, IL 6, IL 10 in the culture supernatants obtained from both patients and controls. The kits were based on the principle of sandwich ELISA which detected the levels through colorimetry and the concentrations were calculated using standard curves obtained in each kit.

### 2.8 F- actin staining

Cultured cells were fixed using 2% PFA for 10 minutes, followed by washing with 1x PBS. They were then treated with 0.1% Triton X-100 for 3–5 minutes. After removing the Triton X-100, fluorescein Phalloidin (ThermoFisher) was added and incubated for 20–30 minutes. Finally, the cells were mounted using DAPI mounting media.

### 2.9 Phagocytosis assay

To assess the effectiveness of phagocytosis in PV, a kit from Cayman chemical Phagocytosis assay kit (#500290) was used and manufacturer’s protocol was followed where latex beads IgG tagged with FITC were used with 1:500 dilution in 1 x 10^6^ cells/well of 6 well plate and were incubated for 4 hours at 37 degrees. The number of beads engulfed was then detected using flow cytometry.

### 2.10 Arginase assay

The Arginase Activity Assay kit (#MAK112 by Sigma) was used for measuring arginase activity in culture. Manufacturer’s protocol was followed. In this assay, arginase facilitates the conversion of arginine to urea and ornithine. The resulting urea reacts specifically with a colour development reagent, producing a coloured product proportional to the arginase activity present. One unit of arginase denotes the amount of enzyme capable of converting 1.0 micromole of L-arginine to ornithine and urea per minute at pH 9.5 and 37 degrees Celsius. The kit boasts a detection limit of 0.3 unit/L for the 2-hour arginase reaction in a 96-well format.

### 2.11 Cytotoxicity assay

A kit provided by Promega- CytoTox 96^®^ Non-Radioactive Cytotoxicity Assay was used. It is a colorimetric based LDH enzyme release detection kit. The target K562 cells number was optimized to 5000 cells/well of 96 well plate. The cultured NK cells from PV and HC were used as effector cells to a standardized E: T (effector: target) ratio of 10:1. The assay was carried out in triplicates and as per manufacturers’ protocol.

### 2.12 Immunofluorescence

Immunofluorescence staining was performed in skin biopsies of 10 PV patients with significant to extensive severity as per PDAI scoring and individuals with non-inflammatory conditions like lipoma as healthy controls embedded in paraffin blocks. The sections were deparaffinized, rehydrated and treated citrate buffer for antigen retrieval. Next, sections were rinsed with 1xPBS and treated with 5% BSA in PBS containing 1% Triton-X100 as a blocking buffer for 1hr at 37 °C. Sections were incubated with primary antibodies- anti- mouse CD68, anti-rabbit iNOS, anti-rabbit CD163 and anti-rabbit CD56 overnight at 4 °C. Next day, sections were rinsed in PBS and treated with fluorophore conjugated secondary antibodies (anti-mouse: DyLightTM 488 conjugate (Invitrogen) and anti- rabbit: DyLightTM 550 conjugate (Invitrogen) to tag primary antibodies. Slides were incubated for 1h at room temperature in dark. Sections were washed with 1X PBS and mounted with Fluoroshield with DAPI and observed under confocal microscope. Images were analysed and quantified using ImageJ/Fiji software (National Institutes of Health, Bethesda, USA).

### 2.13 Statistical analysis

Flow cytometry data was graphically depicted and analysed utilizing FlowJo 10.8 software. All the data was presented as mean ± standard deviation (S.D.). To draw comparisons between patient and control groups for non-parametric data, Wilcoxon rank-sum (Mann-Whitney) test, with statistical significance set at p ≤ 0.05 was used. To compare between groups, two-way ANOVA (Sidak’s/Tukey’s multiple comparison test) was used. Statistical analyses were performed using GraphPad Prism 8.0.

## 3 Results

### 3.1 Increased prevalence of macrophages like monocytes along with increased M1/M2 ratio in circulation

**Figure 1:**
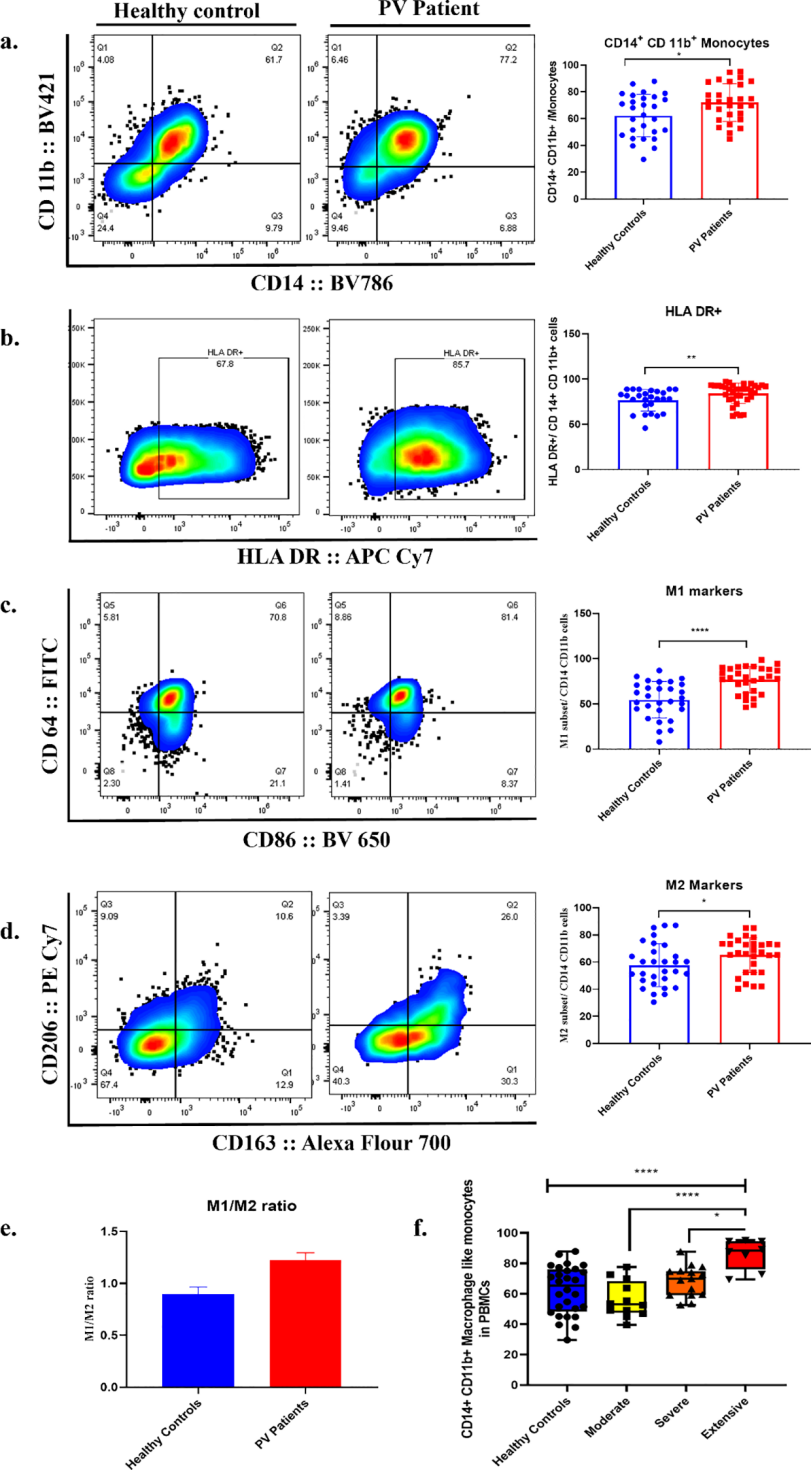
Immunophenotyping of macrophage like monocytes in the peripheral circulation. **(a)** Pseudo colour plot demonstrated the frequency of dual positive CD11b +CD14+ macrophage like monocytes in healthy control and PV patient in PBMCs. Quantification of CD11b +CD14+ cells were demonstrated through bar graph, indicating significant elevation of macrophages in PV. **(b)** HLA DR expression profile in macrophage like monocytes in PBMCs. CD11b +CD14+ macrophage like monocytes were further gated for the subtypes. **(c)** CD64+ CD86+ M1 population **(d)** CD163+ CD206+ M2 population. **(e)** M1/M2 macrophage subset ratio in peripheral circulation. **(f)** The data in box and whisker graph represents correlation of macrophage with disease severity. Mann-Whitney U test was performed to analyse the datasets. Bar graphs were presented in Mean ± SD format. ****p<0.0001, **p<0.005, *p<0.05, n=35.

PBMCs were employed by tagging them with specific antibodies required for the immunophenotyping of macrophage-like monocytes in the peripheral circulation through flow cytometry technique. Upon analysis, a significant increase in the CD11b+ CD14+ monocytic population was observed in the PV patients when compared to healthy controls (*p= 0.04) (**Figure 1a**). Further analysis to investigate the presence of the HLA DR marker, a critical indicator of monocyte activation status, displayed a notable increase in the PV patients (*p = 0.002) (**Figure 1b**). Additionally, to evaluate the possible skewing of macrophage towards either the pro-inflammatory M1 or the anti-inflammatory M2 phenotype, the findings revealed a significant elevation of both M1 markers (CD64 and CD86) (p<0.0001) and M2 markers (CD163 and CD206) (*p= 0.04) in PV (**Figures 1c, d**). Further analysis to determine the M1/M2 ratio highlighted an increase in this ratio in PV patients (1.49 ± 0.62) compared to healthy controls (0.97 ± 0.44), highlighting a possible shift in the macrophage polarization towards a more pro-inflammatory state than a regulatory anti-inflammatory state in Pemphigus Vulgaris (**Figure 1e**). We also correlated the severity of patients with percentage frequency of total macrophage like monocytes (**Figure 1f**). The results showed a significant increase in macrophage population with severity of the patient. Although moderate and severe conditions did not show significant differences with HC, extensive severity showed significant upregulation in macrophage population as shown in **Figure 1**.

### 3.2 Skewing of macrophages towards M2 phenotype *in vitro* (monocyte derived macrophage cultures)

**Figure 2:**
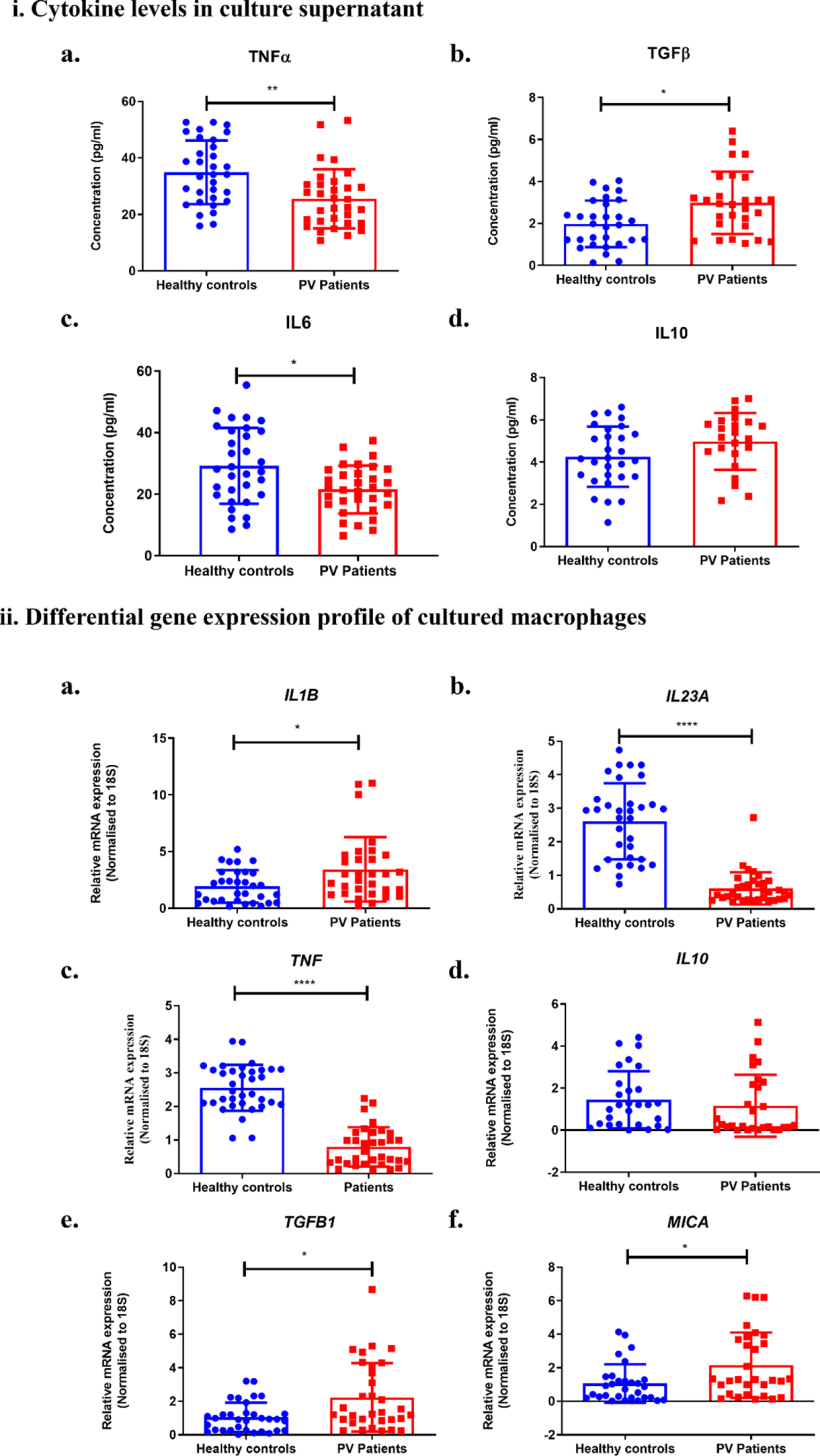
Cytokines in culture supernatants and relative mRNA expression in cultured macrophages. **(i)** Graph depicts the variable cytokine profile of macrophages assessed using ELISA in the culture supernatants. **(a)** TNFα, **(b)** TGFβ, **(c)** IL 6, **(d)** IL 10. **(ii)** Cultured macrophages were harvested to assess differential gene expression of pro-inflammatory markers **(a)** *IL1B*, which was higher **(b)** *IL23A*, **(c)** *TNF*, which were significantly lower to healthy controls and anti-inflammatory markers **(d)** *IL10* and **(e)** *TGF β* which was also higher. **(f)** *MICA* which a ligand for NKG2D was also upregulated. Bar graph representing mean with SD. All values were normalised to housekeeping gene 18S. ****p<0.0001, ***p<0.001, **p<0.005, *p<0.05, n=32.

**Figure 3:**
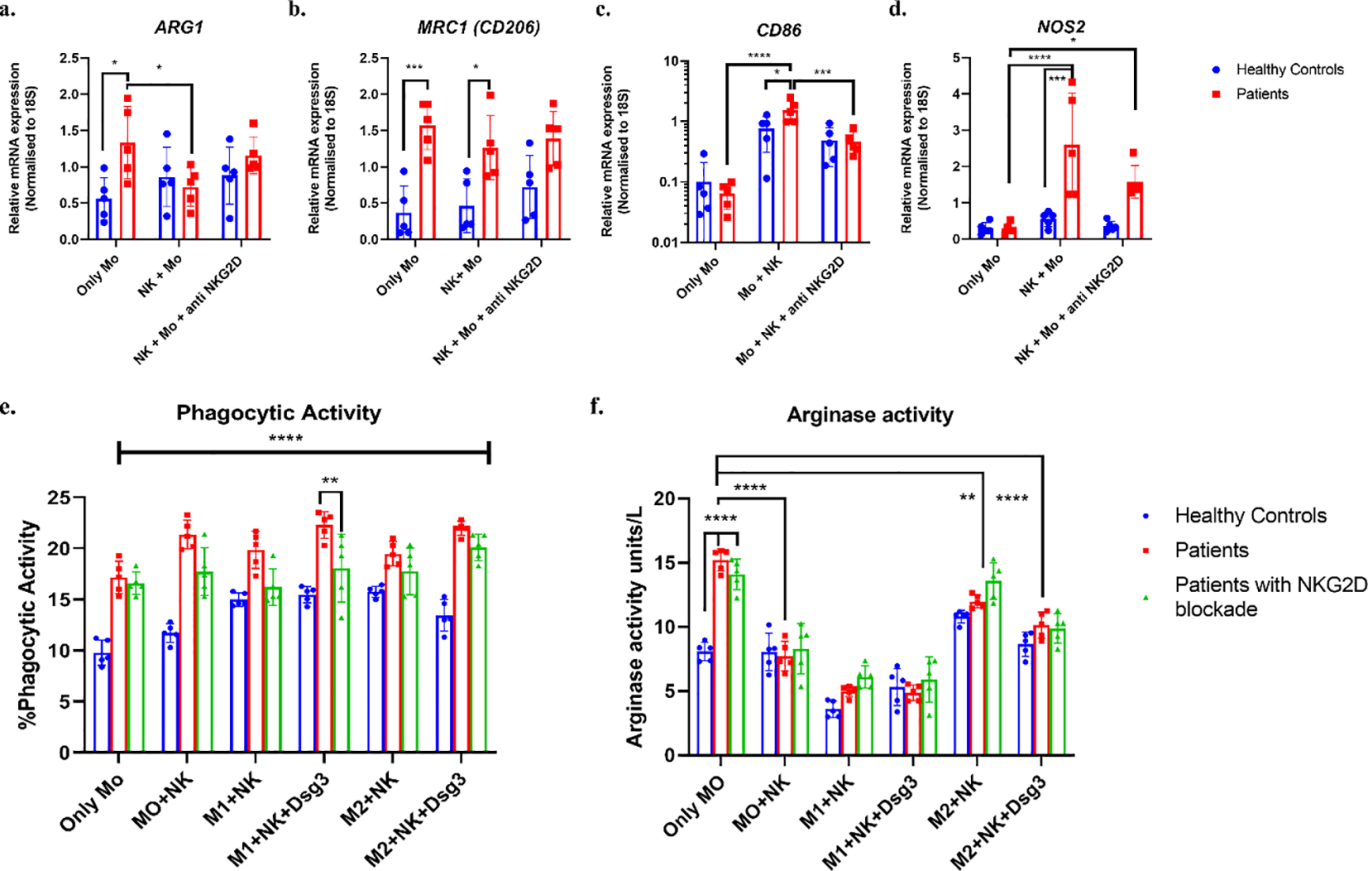
Altered functional markers and activity of macrophages following co-culture with NK cells. Cultured macrophages were harvested before and after co-culture with NK cells to compare the gene expression of M2 related markers **(a)** *ARG1* and **(b)** *CD206* and M1 related markers **(c)** *CD86* and **(d)** *NOS2*. All values were normalised to housekeeping gene 18S. **(e)** Phagocytic activity: Bar graph represents mean with SD for the percent of IgG-FITC beads engulfed by macrophages after 4 hours incubation with 1:500 dilution. **(f)** Arginase activity: Bar graph represents mean with SD for the arginase activity detected in macrophage lysates. ****p<0.0001, ***p<0.001, **p<0.005, *p<0.05, n=05. Condition 1 – without co-culture (Only NK/Only MO), Condition 2- without stimulation co-culture (NK+ MO), Condition 3- M1 macrophages polarized with 10ng/ml IFNγ and 20ng/ml LPS co-culture (NK+M1), Condition 4- M1 macrophages polarized with 10ng/ml IFNγ and 20ng/ml LPS and co-culture stimulated with 10ng/ml Dsg3 (NK+ M1+ Dsg3), Condition 5- M2 macrophages polarized with 10ng/ml IL 4 and 20ng/ml IL 10 co-culture (NK + M2) and Condition 6- M2 macrophages polarized with 10ng/ml IL 4 and 20ng/ml IL 10 and co-culture stimulated with 10ng/ml Dsg3 (NK+ M2+ Dsg3).

Macrophages can produce both pro-inflammatory and anti- inflammatory cytokines. The production of cytokines as well as their plasticity depends on the environmental stimulus. Estimation of cytokines in culture supernatants showed a significant upregulation of anti-inflammatory TGFβ (3.41 ± 1.024 vs 1.92 ± 1.11) alongside significant downregulation of pro-inflammatory TNFα (23.04 ± 7.250 vs 38.52 ± 9.161) and IL 6 (20.47 ± 7.565 vs 28.41 ± 9.873) in the culture supernatant (**Figures 2i, a, b, c**). No such difference was observed in the levels of IL 10 (5.497 ± 1.062 vs 4.738 ± 0.9280). Additionally, cultured macrophages were harvested to synthesize cDNA which was then evaluated for changes in the expression levels of selected pro-inflammatory and anti-inflammatory cytokines along with M1 and M2 specific surface and functional markers to ascertain the cells’ polarity and plasticity. During this analysis, we noted a substantial increase in the M2 related markers’ expression of *TGFB1* (2.31 ± 1.53 vs 1.07 ± 0.87) (**Figures 2ii, e**), *ARG1* (*p<0.01) and *CD206* (***p<0.001) (**Figures 3a, b**) with no significant change in *IL10* levels (2.02 ± 1.31 vs 1.33 ± 1.22) (**Figures 2ii, d**). In contrast, both pro inflammatory cytokines, *IL23A* (0.61 ± 0.49 vs 2.43 ± 1.80) and *TNF* (0.85 ± 0.56 vs 2.69 ± 1.68) exhibited significant reductions along with no change in other M1 markers such as *NOS2* and *CD86* in their expression levels. Intriguingly, the expression of *IL1B* (3.34 ± 2.73 vs 1.96 ± 1.58) was notably elevated in PV (**Figures 2ii, a, b, c**). Additionally, we examined the expression of the NKG2D ligand MHC class I chain-related molecule A (*MICA*), where we observed a significant upregulation (2.19 ± 1.91 vs 1.17 ± 1.09) (**Figures 2ii, f**).

### 3.3 M1 macrophages dominate the lesional sites of pemphigus vulgaris

**Figure 4:**
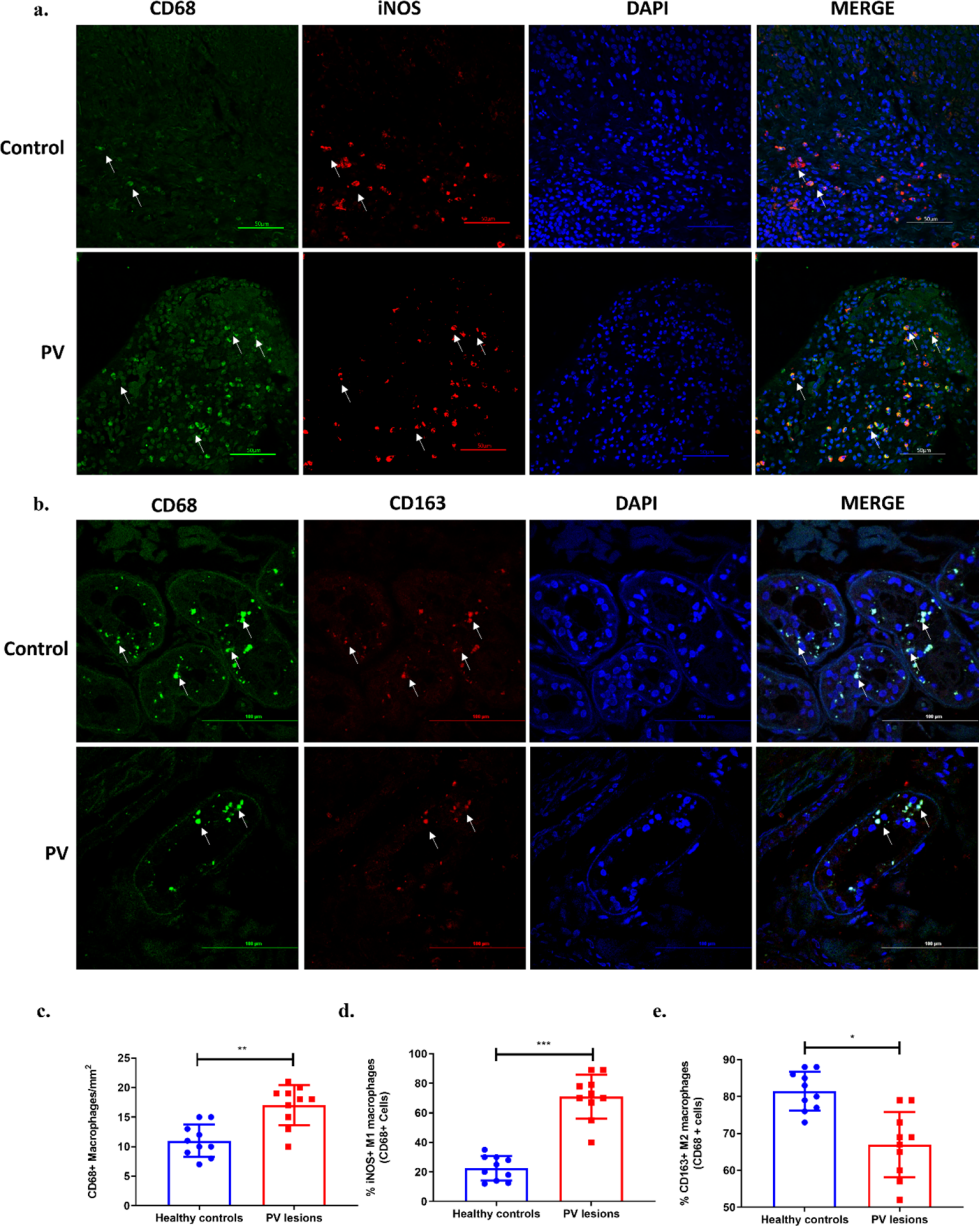
Macrophages and their subtypes in PV lesional tissues. Representative confocal microscopic images of M1 and M2 macrophages in control and PV tissue. **(a)** M1 phenotypic cells are shown as cells which are CD68+ (green) and iNOS+ (red). Tissue sections were stained with primary antibody CD68 conjugated with anti-mouse FITC labelled secondary antibody (green), primary antibody iNOS conjugated with anti-rabbit Alexa fluor 550 labelled secondary antibody (red) and DAPI mounting media =blue. The dilution used for CD68 was standardised at 1:100 and for iNOS at 1:200. Images were captured at 40x magnification. Scale bar= 50µm. **(b)** M2 phenotypic cells are shown as cells which are CD68+ (green) and CD163+ (red). Tissue sections were stained with primary antibody CD68 conjugated with anti-mouse FITC labelled secondary antibody (green), primary antibody CD163 conjugated with anti-rabbit Alexa fluor 550 labelled secondary antibody (red) and DAPI mounting media =blue. The dilution used for CD163 was standardised at 1:200. Images were captured at 60x magnification. Scale bar= 100µm. **(c–e)** Column bar graph representing MFI (mean fluorescent intensity) of macrophages and their subtypes calculated using ImageJ software. ***p<0.001, **p<0.005, *p<0.05, n= 10.

Immunofluorescence was employed to detect macrophages within the lesioned tissues from biopsies of patients. The staining process highlighted the presence of macrophages, identified by the CD68+ pan marker, predominantly in the dermis layer of the skin, where both M1 and M2 macrophage subtypes were also delineated (**Figure 4**). A significant rise in the population of M1 macrophages, marked by CD68+ iNOS+ markers, was noted within the lesions of PV patients (75.57 ± 12.53) relative to the controls (23.50 ± 8.98), pointing to their substantial upregulation in the affected tissue (**Figures 4a, d**). Additionally, the M2 macrophage phenotype, marked by CD68+ CD163+ markers, was found to be significantly decreased in the lesions of pemphigus vulgaris (PV) patients (67.6 ± 9.19) when compared to healthy controls (81.60 ± 6.43) (**Figures 4b, e**). In summary, there was a significant elevation in the overall population of CD68+ macrophages in PV lesions (18.17 ± 2.78) relative to those in healthy individuals (11.17 ± 2.64) (**Figure 4c**).

### 3.4 Possible interaction between NK cells and macrophages through NKG2D: MICA/B, F-actin and colocalization

**Figure 5:**
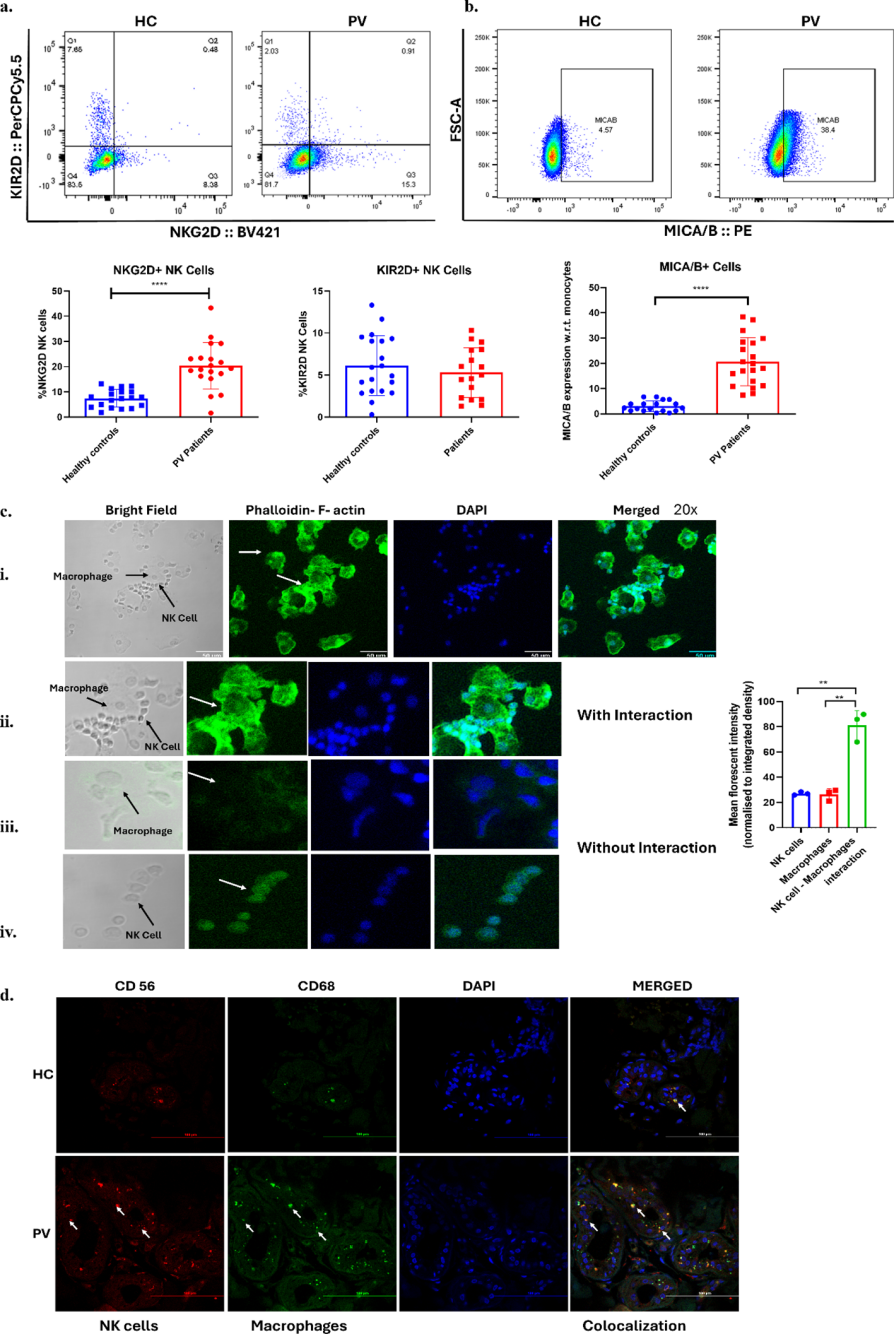
Interaction between NK cells and macrophages. **(a)** Estimation of NK receptors: Bar graph represents mean with SD for percentage of NKG2D+ and KIR2D+ NK cells in PBMCs of PV patients. **(b)** Expression of NKG2D ligand MICA/B: Bar graph represents mean with SD of percent MICA/B+ monocytes in PBMCs. ****p<0.0001, n=20. **(c)** i) Accumulation of F- actin accumulation can be observed at the site of interaction between NK cells and macrophages forming a ring like structure at 2.58 times zoomed 20x magnification. ii.) Zoomed-in 20x images representing higher F-actin at the site of conjugation/interaction. iii.) and iv.) Zoomed-in 20x images representing lower F- actin in unconjugated macrophages and NK cells respectively. Bar graph represents mean with SD for mean florescent intensity of F- actin tagged with phalloidin. The mean values were normalized to integrated density values. **p<0.005. **(d)** Representative confocal microscopic images of NK cells and macrophages colocalization in PV lesional tissue. NK cells are shown as cells which are CD56+ (red) and macrophages as CD68+ (green). Tissue sections were stained with primary antibody CD68 conjugated with anti-mouse FITC labelled secondary antibody (green), primary antibody CD56 conjugated with anti-rabbit Alexa fluor 550 labelled secondary antibody (red) and DAPI mounting media =blue. The dilution used for CD56 was standardised at 1:100 and for CD68 at 1:200. Images were captured at 60x magnification. Scale bar= 100µm.

In our previous study, we noted significant upregulation of mRNA expression of NK cells surface receptors- NKG2D and KIR2D (6). In the current study, we observed a significant increase in NKG2D expression on NK cells in PV (20.34 ± 9.25) compared to controls (7.42 ± 3.46) while no such difference was observed in KIR2D (5.29 ± 2.93 vs 6.12 ± 3.55) in the periphery (**Figure 5a**). Additionally, we observed a significant upregulation in its ligand i.e. MICA/B on macrophages in PV (20.61 ± 9.50) compared to controls (3.01 ± 2.18) (**Figure 5b**). These results were in concordance with the mRNA expression of MICA/B observed in **Figures 2ii, f**. This upregulation of this receptor-ligand duo suggests their involvement in interaction. The interaction between NK cells and macrophages also requires physical modulations such as cytoskeletal changes during the immune response phase. F-actin or filamentous actin is a major component of cytoskeleton involved in various processes such as cell mobility, maintenance of cell structure, formation of phagocytic cups in macrophages and so on. Upon co-culture, we observed notable accumulation of F actin around the site of interaction compared to unconjugated/without interacting sites of NK cells and macrophages (**p= 0.0014 and 0.0017), respectively (**Figure 5c**). Furthermore, the immunofluorescence staining revealed that NK cells and macrophages, characterized by CD56+ and CD68+ markers respectively, share similar localization and were present in the dermis layer of the skin (**Figure 5d**). The pronounced presence of NK cells and macrophages together and increased infiltration were observed in the affected areas of PV patients, as opposed to the control samples, underscores their significant upregulation in the diseased tissue. The detailed localization provides valuable insights into the cellular dynamics at play in the immunological landscape of pemphigus vulgaris.

### 3.5 Impact of NK cells - macrophage interactions on their functional activities

#### 3.5.1 Co-culture with NK cells induced changes in macrophage functional markers

To assess the influence of NK cells on the gene expression of functional markers in macrophage subtypes, macrophages were co-cultured with NK cells. Gene expression analysis showed a significant upregulation of M1-associated markers, including *NOS2* (***p<0.001) and the surface marker *CD86* (***p<0.001), indicating enhanced inflammatory and antigen-presenting capacity (**Figures 3c, d**). These changes were not present prior to co-culture, suggesting that the observed shift toward M1 polarization is a direct consequence of NK cell interaction. Conversely, the M2-associated marker *ARG1* (*p<0.01) was significantly downregulated, implying a reduced capacity for tissue repair and immune regulation. *MRC1* (*CD206)* expression remained unchanged, suggesting that M2 polarization was only partially reprogrammed upon NK cell exposure (**Figures 3a, b**).

Furthermore, we used anti NKG2D antibody to block NKG2D on NK cells for co-culture (**Supplementary Figure 4**). We observed non-significant increase in *ARG1* and *CD206* (**Figures 3a, b**), however, CD86 was significantly reduced compared to condition without blocking (***p<0.001) (**Figure 3c**). *NOS2* expression was non significantly reduced after NKG2D blockade (**Figure 3d**).

#### 3.5.2 Disparity in the phagocytosis activity in macrophages obtained upon co-culture

The percent phagocytosis in PV was significantly upregulated compared to healthy controls in all conditions except condition 5 where M2 stimulations were given without Dsg3, suggesting highly effective macrophage activity in diseased condition (**Figure 3e**). In healthy controls, macrophages showed higher phagocytic activity when co-cultured with NK cells under M1 stimulation in both condition 3 (*p<0.01) and condition 4 (**p<0.008) and interestingly in condition 5 (**p<0.004) under M2 stimulation compared to condition 1.

In PV, phagocytic levels were particularly higher in condition 4 (**p<0.006) and condition 6 (**p<0.007) where Dsg3 antigen was given compared to condition 1. After NKG2D blockade, there was non-significant reduction in phagocytic activity under all conditions except condition 4 under M1+ Dsg3 stimulation, which demonstrated significant downregulation when compared to without blockade levels (**p<0.008).

#### 3.5.3 Disruption in arginase activity of macrophages in PV

Following co-culture, macrophages were harvested and lysed to assess the impact of NK cells on M2-associated arginase activity. Notably, arginase activity was highest when macrophages were cultured in isolation but significantly decreased after co-culture with NK cells under condition 2 (**p<0.005) (**Figure 3f**). Although arginase activity was modestly elevated in the M2-polarizing condition (Condition 5) (**p<0.005), the increase was not statistically significant. Interestingly, in Condition 6, which included both M2 stimulation and Dsg3 exposure, a significant reduction in arginase activity was observed (*p<0.05), suggesting a potential but yet unidentified role of Dsg3. We speculate that increased disease severity in pemphigus vulgaris may influence macrophage polarization. Overall, these findings indicate that M2-associated arginase activity is impaired when macrophages are co-cultured with NK cells, particularly in the PV context. However, post NKG2D blockade, levels were increased non significantly under M2 state (condition 5), suggesting partial involvement of NKG2D in interaction.

#### 3.5.4 Increased activation of NK cells detected via CD69

**Figure 6:**
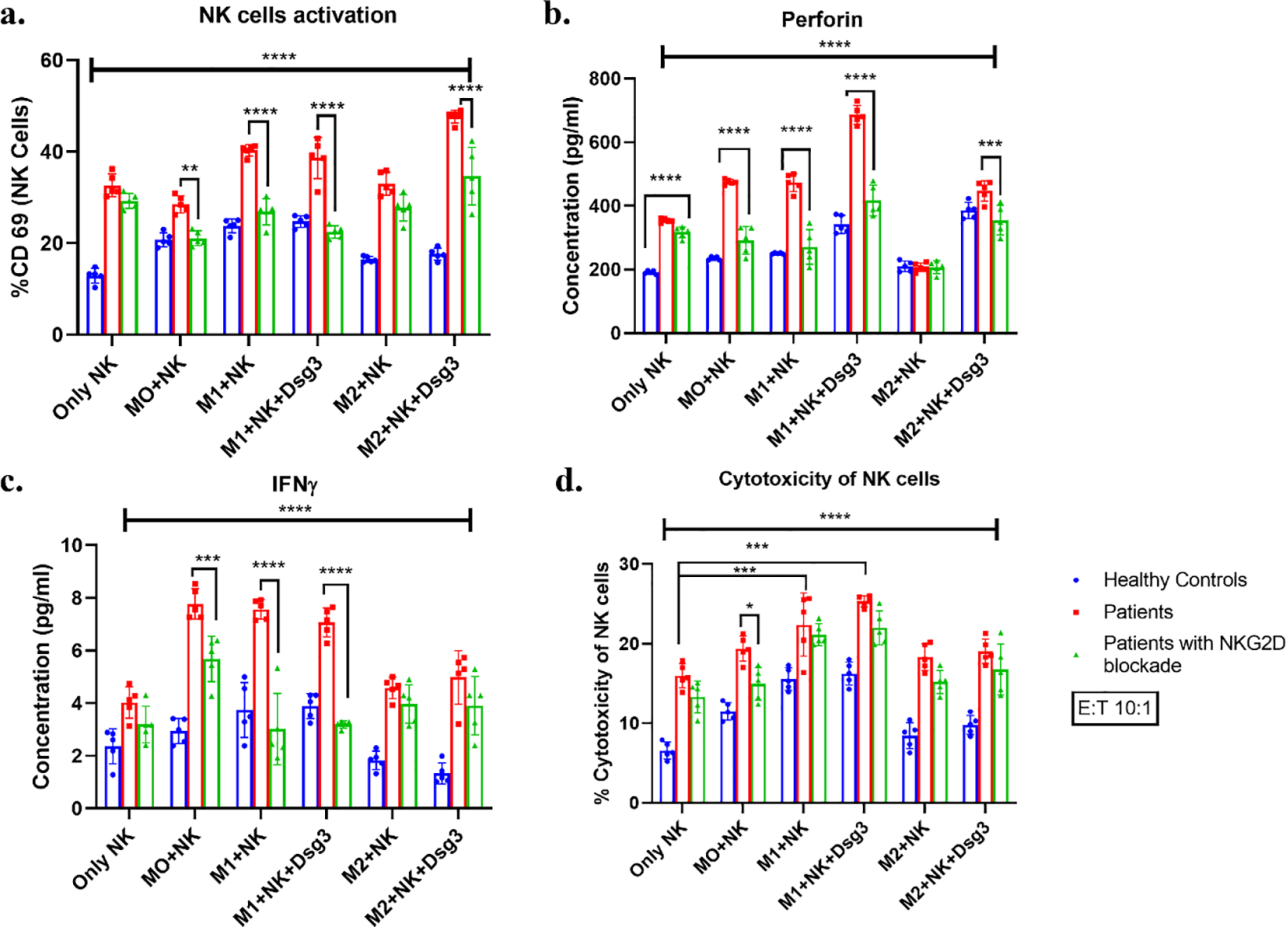
Impact of co-culture on the functional activities of NK cells under different stimulatory conditions. **(a)** CD69 as an activation marker was used to assess NK cell activation. **(b)** and **(c)** Perforin and IFNγ levels were assessed using ELISA in the culture supernatants respectively. **(d)** Mean with SD of % cytotoxicity of NK cells at 10:1 E:T ratio where target K562 was optimized to 5000cells/well of 96 well plate. *p<0.05, **p<0.005, ***p<0.001, ****p<0.0001. n=05. Condition 1 – without co-culture (Only NK/Only MO), Condition 2- without stimulation co-culture (NK+ MO), Condition 3- M1 macrophages polarized with 10ng/ml IFNγ and 20ng/ml LPS co-culture (NK+M1), Condition 4- M1 macrophages polarized with 10ng/ml IFNγ and 20ng/ml LPS and co-culture stimulated with 10ng/ml Dsg3 (NK+ M1+ Dsg3), Condition 5- M2 macrophages polarized with 10ng/ml IL 4 and 20ng/ml IL 10 co-culture (NK + M2) and Condition 6- M2 macrophages polarized with 10ng/ml IL 4 and 20ng/ml IL 10 and co-culture stimulated with 10ng/ml Dsg3 (NK+ M2+ Dsg3).

After co-culture experiments, NK cells in suspension were collected, stained with the CD69 activation marker, and analyzed via flow cytometry (**Supplementary Figure 5**). The activation of NK cells in PV patients was notably higher compared to healthy individuals, regardless of stimulatory conditions (**Figure 6a**). In the healthy state, co-culturing NK cells with macrophages, even without specific stimuli (condition 2) (*p<0.02), led to a significant increase in CD69 expression on NK cells. Activation levels rose further in the M1 state following stimulation with LPS and IFNγ, regardless of the presence of Dsg3 {conditions 3 (**p<0.001) and 4 (***p<0.001)}, indicating that NK cell activation occurs in the presence of macrophages, especially of the M1 phenotype. Conditions 3 (M1) demonstrated significant activation compared to condition 5 (*p<0.01), but not to condition 6, highlighting the role of Dsg3 in enhancing NK cell activation in the M2 phenotype in a healthy state.

In PV cases, significant NK cell activation was observed with M1 stimulation {condition 3 (*p<0.02) and condition 4 (*p<0.04)} and M2 stimulation where the PV antigen Dsg3 was present (condition 6 (*p<0.004). Furthermore, activation increased in M1 stimulations with or without Dsg3 (conditions 3 and 4 (***p<0.0003, ***p<0.0005)) compared to condition 2, with a remarkable increase in condition 6 (***p<0.0001) over condition 2. Additionally condition 6 (**p<0.004) was significantly upregulated to condition 1, suggesting that Dsg3 might influence macrophage polarization or plasticity towards the M1 phenotype, thereby enhancing NK cell activation. This was further validated using NKG2D blocking, the activation of NK cells was reduced post blockade under all conditions. The expression of CD69 was significantly lowered in case of condition 2 (**p= 0.001) and M1 stimulated conditions 3 (****p< 0.0001) and 4 (****p< 0.0001) along with condition 6 (***p< 0.0001) compared to conditions without NKG2D blocking.

#### 3.5.5 Elevated cytotoxicity of NK cells after co-culturing with macrophages

NK cells harvested from PV patients showed greater lysis of K562 displaying higher cytotoxicity to healthy controls irrespective of stimulatory conditions (**Figure 6d**). In healthy conditions, *in vitro* interaction led to an increase in NK cells’ cytotoxicity (condition 2) (*p<0.02) with significant increases in M1 stimulations i.e. conditions 3 (***p<0.0002) and 4 (****p<0.0001) compared to condition 1. Cytotoxicity was much higher when Dsg3 was present in M1 stimulation compared to condition 2. Condition 3 and condition 4 led to notably higher cytotoxicity compared to M2 stimulations both in condition 5 (**p<0.006) and condition 6 (*p<0.01).

In PV, there was no increase in cytotoxicity in presence of macrophage alone i.e. condition 2 when compared to condition 1. However, when M1 stimulations were provided i.e. condition 3 (***p<0.0004) and 4 (***p<0.0002), a significant increase in cytotoxicity was seen which was in concordance with the NK cells’ activation status compared to condition 2. The elevation in M1 stimulations was much higher compared to M2 stimulations i.e. condition 5 and condition 6. On NKG2D blocking, cytotoxicity reduced non significantly in all conditions but condition 2 (*p= 0.03) displayed significant reduction compared to others and it’s no blocking counterpart.

#### 3.5.6 Altered perforin levels in culture supernatants of PV

Co-culture supernatants from all the stimulating conditions were used to assess perforin levels which is a characteristic secretion of NK cells. Results obtained displayed significant upregulation in PV irrespective of the stimulatory conditions except for condition 5 which showed no such difference (**Figure 6b**).

In healthy controls, perforin levels were interestingly higher in condition 4 (****p<0.0001) and condition 6 (****p<0.0001) where Dsg3 was added compared to condition 1. Whereas in PV, perforin levels rose under co-culture conditions in condition 2 (***p<0.0005), in M1 stimulations, condition 3 (***p<0.0003) and condition 4 (****p<0.0001) and condition 6 (**p<0.003) compared to condition 1. Interestingly, M2 stimulations showed lower levels of perforin compared to all other conditions but not to healthy controls suggesting insufficient regulation by M2 phenotype. This was further validated when NKG2D was blocked, there was significant reduction of perforin in M2 condition 6 (p=0.0004) and levels were comparable to healthy control in condition 5. In addition to this, perforin significantly reduced in condition 2 (****p<0.0001), condition 3 (****p<0.0001) and condition 4 (****p<0.0001), suggesting critical involvement of NKG2D in NK macrophage interaction. However, reduction did not reach the levels of healthy control indicating presence of other factors in their interaction.

#### 3.5.7 Increased IFNγ secretion by NK cells after co-culture

Along with perforin, culture supernatants were used to assess IFNγ levels secreted by NK cells (**Figure 6c**). Irrespective of culture conditions, IFNγ levels were higher in PV compared to healthy controls.

In PV, IFNγ levels were increased when NK cells were co cultured with macrophages (condition 2, ****p<0.0001) particularly in presence of M1 stimulations irrespective of Dsg3 i.e. condition 3 (****p<0.0001) and 4 (****p<0.0003) respectively compared to condition 1. Condition 5 and condition 6 showed lower levels of IFNγ compared to other conditions, suggesting insufficient regulation of NK cell’s IFNγ secretion by M2 phenotype.

When NKG2D was blocked, there was downregulation in IFNγ secretion especially in conditions 2 (****p<0.0001), 3 (****p<0.0001) and 4 (****p<0.0001).

## 4 Discussion

Natural killer cells and macrophages are the key innate components of our immune system. Individually their role and characterization have been widely studied across different diseases and pathogenesis (45–48). However, their interaction with each other remains elusive and atypical (38). In our previous study, we characterized the role of NK cells in pemphigus vulgaris. We reported an increase in NK cells frequency. Our study further provided a deep insight into the functioning of NK cells which showed increased effector activity, significant increase of NK cells transcription factors and activating receptors as well as release of proinflammatory IFNγ in pemphigus vulgaris. Increased infiltration of NK cells in lesional tissues suggested their involvement in blister formation (6). But macrophages and their contribution to PV remain unexplored. However, there are some studies suggesting their presence in lesional tissues and keratinocyte clefts but their significance, number, phenotypic and functional characteristics remained unknown (16–18, 49). In this study, we provided evidence based on phenotypic and functional relevance of innate immune axis contributed by critical coordination by NK cells and macrophages, that may contribute to the immunopathogenesis of PV.

We observed significant elevation in CD14+ CD11b+ macrophage-like monocytes as well as in M1 associated markers (CD64 and CD86) and M2 like macrophages (CD163 and CD206), indicating the dynamic and heterogeneous nature of macrophages in PV patients. Additionally, M1 macrophages with Dsg3 antibody titres displayed positive and significant correlation while M2 macrophages displayed weak and non-significant negative correlation (**Supplementary Figures 9a, b**). While this finding was presented in dichotomous M1 like and M2 like phenotype, it is critical to understand the wide range of diverse phenotypes attributed by macrophages under *in vivo* state. They exhibit substantial plasticity and overlapping properties depending on their environmental cues that do not strictly follow M1 and M2 polarization states but M1 like and M2 like states. Therefore, in autoimmunity, it is widely reported as a ratio of balance between M1/M2 that could contribute to disease pathogenesis and provide functional trends acknowledging macrophage complexities (50–52). Subsequently, an increased M1/M2 ratio was observed in this study, suggesting a bias of macrophages towards a pro-inflammatory nature, might contributing to inflammation in PV. Increased monocyte/macrophage populations have been noted in many autoimmune conditions, contributing to inflammation and tissue remodelling (35, 53–56). Furthermore, chronic autoimmune conditions have demonstrated the presence of both macrophage subsets maintaining a balance, suggesting their role in both pathogenic and protective functions (35). Furthermore, analysis of cytokine and mRNA levels in macrophage culture supernatants revealed a significant increase in the anti-inflammatory cytokines *TGFB1*, M2 markers- *ARG1* and *CD206*, coupled with a notable decrease in the pro-inflammatory cytokines *IL23*, *TNF* and *IL6* with no significant difference in expression levels of *NOS2* and *CD86*. These results show a predominance of the M2 phenotype *in vitro*, characterized by increased arginase which may cause suppression of nitric oxide, thereby promoting anti-inflammatory responses in addition to tissue repair supported by enhanced *CD206* expression, hence, indicating their biological significance in PV pathogenesis (57). Moreover, NKG2D ligand *MICA*, showed a significant upregulation in its mRNA levels. These heightened functional markers further support the results, demonstrating the activation of both M1 and M2 macrophages concurrently in PV. In concordance to SLE, PV exhibits a role of both M1 and M2 phenotypes, where M1 promotes inflammation, and this increased inflammation is counterbalanced by the M2 phenotype under *in vitro* conditions (58).

We observed an increased infiltration of CD68+ macrophages in the dermis of PV tissues. However, M1 macrophages showed a significant increase in PV tissues while M2 macrophages exhibited a significant decrease. Our findings were consistent with several other autoimmune disorders such as vitiligo and psoriasis, but they exhibited differences compared to conditions like bullous pemphigoid and systemic sclerosis (59–62). We hypothesize that this decrease in M2 macrophages could be attributed to the inflammatory conditions within the tissue, a notion further supported by the relative mRNA expression analysis in tissues. This analysis revealed a significant upregulation of pro-inflammatory markers such as *TNF, IL1B, and IL23* in PV tissues compared to healthy controls (**Supplementary Figure 6**). Previous reports have suggested *IL23* and *IL6* promote Th17 responses by maintenance and differentiation of Th 17 cells and reinforcing a positive loop between Th17 and inflammatory macrophages (63–65). Given that PV has been reported as Th17 driven autoimmune disease, the presence of increased inflammatory M1 like macrophages as reported in this study may represent a key contributor in amplification of inflammatory responses. Furthermore, *IL1B* is a potent pro inflammatory cytokine released by macrophages under pathogenic conditions via involvement of inflammasome signalling, however, whether the increased *IL1B* observed in the study depends on inflammasome needs further investigation (66). Conclusively, the results indicate an increase in both M1 like and M2 like macrophages in PV. However, while there was a predominance of the M2 phenotype observed *in vitro*, the patients result suggest a contrasting predominance of the M1 phenotype.

Hence, to investigate whether this polarization of macrophages could be influenced by interactions with other immune cells. We were the first to delve into exploring the interplay between NK cells and macrophages in the context of autoimmune disorders. As observed, the relative mRNA levels of surface receptors *KIR2D* and *NKG2D* on NK cells (6), as well as *MICA/B* on macrophages, showed significant upregulation. Our findings revealed a significant upregulation of NKG2D+ NK cells and their ligand MICA/B+ macrophages in peripheral circulation also. They also showed positive and significant correlation (**Supplementary Figure 9e**). Additionally, MICA/B was significantly correlated with disease burden as assessed by Dsg3 antibody titres (**Supplementary Figure 9d**). The heightened levels of NKG2D and MICA/B suggest their involvement in interactions. These results are supported by a report in cutaneous lupus erythematosus where this receptor ligand duo resulted in increased susceptibility through increased cytotoxicity and IFNγ by NKG2D+ NK cells (67). Furthermore, the localization patterns of CD68+ macrophages and CD56+ NK cells, were significantly increased in PV lesioned tissues compared to controls. Additionally, we demonstrated synaptic re-localization during cell-to-cell contact using F-actin staining, revealing an increased accumulation of F-actin staining at the interaction site compared to when cells were not in contact with each other. The relocation of F-actin during cell-to-cell contact has been well-documented in previous studies, indicating its role in maintaining cell contact and facilitating cell mobility, migration, and integrity (68).

This study delved deeper into exploring the outcomes of the interaction between NK cells and both polarized and unpolarized macrophages, in the presence or absence of the PV autoantigen Dsg3. The activation status of NK cells was significantly heightened in PV. Interestingly, this activation was further amplified when NK cells were co-cultured with macrophages, particularly under M1 conditions. Moreover, the co-culture led to an increase in NK cell cytotoxicity, IFNγ production, and perforin secretion. The stimulations resembling M1 conditions further bolstered the functional activity of NK cells, enhancing their activation, cytokine production, and cytotoxicity (69). However, M2 polarization tended to diminish the activity of NK cells, in terms of IFNγ production and perforin secretion, their activation status and cytotoxicity. These findings suggest the role of M2 polarization in regulating the inflammation caused by NK cells. However, it also indicates that M2 polarization alone is insufficient to fully suppress NK cell activity and promote anti-inflammatory and repair activities. This was further corroborated by assessing arginase activity, where the M2 phenotype did not show a significant increase. In comparison to other stimulatory conditions, their arginase activity appeared impaired, as M2 macrophages were unable to show sufficient activity. Interestingly, when M2 macrophages were provided with the Dsg3 autoantigen, they tend to promote NK cell activation, perforin secretion, and exhibited significantly higher phagocytic activity, similar to what M1 macrophages displayed in the presence of Dsg3 compared to other stimulatory conditions. This suggests some influence of Dsg3 on macrophage function which needs further clarification. We speculate that there is a polarization of the M2 phenotype towards M1 displaying increased plasticity in the presence of excess autoantigen, reflecting the PV condition, which could explain why M2 macrophages seemed to function inadequately in PV immunopathogenesis. Additionally, following co-culture, macrophages exhibited increased expression of M1 markers- *NOS2* and *CD86* and decreased expression of M2 markers - *CD206* and *ARG1*. This further supports our observation that NK cells drive macrophage polarization toward the M1 like phenotype in PV.

Notably, NKG2D blockade on NK cells resulted in partial but significant decrease in NK cells activity including activation, perforin and IFNγ release along with cytotoxicity. The reduction were prominent particularly in M1 state where expression levels were highly elevated prior to blockade suggesting NKG2D as a key contributor in inflammation. Additionally, M1 like markers *NOS2* and *CD86* were reduced and arginase activity non significantly improved under M2 state post blockade. This observation suggests critical role of NKG2D in NK cell and macrophage interactions, however, also highlights role of contributory factors that compensate for the loss of NKG2D signalling post blockade resulting in persistent expression of functional markers.

Despite these insights, our study present certain limitations. The observed results are primarily based on cross sectional study design, therefore, investigation in longitudinal settings will provide clarity on disease recurrence and temporal dynamics. High dimensional techniques such as single cell transcriptomics and multiplex imaging are required to assess macrophage diversity in detail which may help in providing clarity of the *in vivo* complexities. Although previous studies have demonstrated NK macrophage interactions are primarily cell-cell mediated, presenting more robust functional modulations compared to soluble mediators but differential functional consequences of these need further investigation in PV (70). Additionally, downstream signalling pathways involved in pathogenesis will provide a comprehensive understanding of PV pathology.

This maiden study marks a significant step forward in comprehending the complex bidirectional interactions between NK cells and macrophages in PV. It underscores the crucial role of communication within the immune system. While NK cells exhibit full activity, functionality, and pro-inflammatory effects in PV, macrophages demonstrate a dual role. M2 macrophages show predominance in PV under *in vitro* conditions, but their functionality appears compromised. Instead, there is a prevalence of M1 macrophages at both circulatory and tissue levels. This compromised functionality seems to stem from the crosstalk with NK cells as validated by blocking one of the key markers NKG2D showing partial but significant response, suggesting multifactorial involvement in NK macrophage crosstalk. Therefore, by either promoting M2 polarisation to promote the regulatory cell type by techniques like adoptive transfer therapy or by restraining NK cell functioning, this approach holds translational value as an emerging therapeutic intervention for PV. Furthermore, our study has provided a deeper insight into the complex immune network of PV which might help with the future therapeutics.

## Data Availability

The raw data supporting the conclusions of this article will be made available by the authors, without undue reservation.

## References

[1] Heckler I Hong M Amart Sinha A Venkataraman I . Serological biomarkers and their detection in autoimmune bullous skin diseases. Dermatol Pract Concept. (2022) 12:e2022116. doi: 10.5826/dpc.1202a116. PMID: 35646449 PMC9116534

[2] Melchionda V Harman KE . Pemphigus vulgaris and pemphigus foliaceus: an overview of the clinical presentation, investigations and management. Clin Exp Dermatol. (2019) 44:740–6. doi: 10.1111/ced.14041. PMID: 31378971

[3] Sar-Pomian M Rudnicka L Olszewska M . The significance of scalp involvement in pemphigus: a literature review. BioMed Res Int. (2018) 2018:6154397. doi: 10.1155/2018/6154397. PMID: 29770335 PMC5889856

[4] Porro AM Seque CA Ferreira MCC Simões MM Enokihara S . Pemphigus vulgaris*. Anais Brasileiros Dermatologia. (2019) 94:264–78. doi: 10.1590/abd1806-4841.20199011. PMID: 31365654 PMC6668932

[5] Immunobullous Diseases - Rook’s Textbook of Dermatology - Wiley Online Library. Available online at: https://onlinelibrary.wiley.com/doi/abs/10.1002/9781444317633.ch40?msockid=021b11ab97c368370f360347963169c9 (Accessed March 11, 2026).

[6] Hooda V Khandpur S Arava S Sharma A . Distorted frequency and functionality of natural killer cells in pemphigus vulgaris: a potential therapeutic target. Immunol Lett. (2024) 269:106900. doi: 10.1016/j.imlet.2024.106900. PMID: 39032911

[7] Hooda V Sharma D Singh A Das D Gupta S Arava S . Alterations in chemokine receptor-ligand interactions and costimulatory molecules in DC-NK crosstalk: a novel therapeutic approach for pemphigus vulgaris. Immunol Lett. (2025) 276:107055. doi: 10.1016/j.imlet.2025.107055. PMID: 40582577

[8] Hooda V Khandpur S Sharma A . Augmented IFNγ producing ILC1 and IL 17 producing ILC3 in pemphigus vulgaris: plausible therapeutic target. Cell Immunol. (2025) 408:104910. doi: 10.1016/j.cellimm.2024.104910. PMID: 39718308

[9] Das D Singh A Antil PS Sharma D Arava S Khandpur S . Distorted frequency of dendritic cells and their associated stimulatory and inhibitory markers augment the pathogenesis of pemphigus vulgaris. Immunol Res. (2020) 68:353–62. doi: 10.1007/s12026-020-09166-0. PMID: 33184735

[10] Das D Anand V Khandpur S Sharma VK Sharma A . T helper type 1 polarizing γδ T cells and scavenger receptors contribute to the pathogenesis of pemphigus vulgaris. Immunology. (2018) 153:97–104. doi: 10.1111/imm.12814. PMID: 28815581 PMC5721249

[11] Das D Arava S Khandpur S Santosh KV Akhtar S Sharma A . Dominance and improved survivability of human γδT17 cell subset aggravates the immunopathogenesis of pemphigus vulgaris. Immunol Res. (2024) 72:72–81. doi: 10.1007/s12026-023-09413-0. PMID: 37620509

[12] Satyam A Khandpur S Sharma VK Sharma A . Involvement of T(H)1/T(H)2 cytokines in the pathogenesis of autoimmune skin disease-pemphigus vulgaris. Immunol Invest. (2009) 38:498–509. doi: 10.1080/08820130902943097. PMID: 19811408

[13] Asothai R Anand V Das D Antil PS Khandpur S Sharma VK . Distinctive Treg associated CCR4-CCL22 expression profile with altered frequency of Th17/Treg cell in the immunopathogenesis of pemphigus vulgaris. Immunobiology. (2015) 220:1129–35. doi: 10.1016/j.imbio.2015.06.008. PMID: 26093920

[14] Das D Akhtar S Kurra S Gupta S Sharma A . Emerging role of immune cell network in autoimmune skin disorders: an update on pemphigus, vitiligo and psoriasis. Cytokine Growth Factor Rev. (2019) 45:35–44. doi: 10.1016/j.cytogfr.2019.01.001. PMID: 30773437

[15] IADVL Textbook of Pemphigus and other Autoimmune Bullous Diseases. Available online at: https://jaypeebrothers.com/products/9789356961449 (Accessed June 7, 2024).

[16] Fujimura T Kakizaki A Furudate S Aiba S . A possible interaction between periostin and CD163+ skin-resident macrophages in pemphigus vulgaris and bullous pemphigoid. Exp Dermatol. (2017) 26:1193–8. doi: 10.1111/exd.13157. PMID: 27501402

[17] Furudate S Fujimura T Kambayashi Y Kakizaki A Aiba S . Comparison of CD163+ CD206+ M2 macrophages in the lesional skin of bullous pemphigoid and pemphigus vulgaris: the possible pathogenesis of bullous pemphigoid. Dermatology. (2014) 229:369–78. doi: 10.1159/000365946. PMID: 25401296

[18] Honma T Saito T . Role of macrophages in intraepidermic vesiculation in pemphigus vulgaris. Electron-microscopic observations. Dermatologica. (1979) 159:145–50. doi: 10.1159/000250576. PMID: 478051

[19] Sharygin D Koniaris LG Wells C Zimmers TA Hamidi T . Role of CD14 in human disease. Immunology. (2023) 169:260–70. doi: 10.1111/imm.13634. PMID: 36840585 PMC10591340

[20] Gordon S Taylor PR . Monocyte and macrophage heterogeneity. Nat Rev Immunol. (2005) 5:953–64. doi: 10.1038/nri1733. PMID: 16322748

[21] Veillette A Li J Galindo CC Davidson D Tang Z . Targeting phagocytosis checkpoints for cancer immunotherapy. Nat Rev Cancer. (2026) 26:185–99. doi: 10.1038/s41568-025-00893-w. PMID: 41360986

[22] Sain N Hooda V Singh A Gupta S Arava S Sharma A . Macrophage inhibitory factor alters the functionality of macrophages and their involvement in disease pathogenesis of active generalized vitiligo patients. Cytokine. (2024) 176:156516. doi: 10.1016/j.cyto.2024.156516. PMID: 38340551

[23] Singh A Raja D Kaushal S Seth A Singh P Sharma A . Phenotypic characterization of tumor associated macrophages and circulating monocytes in patients with urothelial carcinoma of bladder. Immunol Res. (2025) 73:66. doi: 10.1007/s12026-025-09624-7. PMID: 40195201

[24] Higashi-Kuwata N Jinnin M Makino T Fukushima S Inoue Y Muchemwa FC . Characterization of monocyte/macrophage subsets in the skin and peripheral blood derived from patients with systemic sclerosis. Arthritis Res Ther. (2010) 12:R128. doi: 10.1186/ar3066. PMID: 20602758 PMC2945018

[25] Ishikawa O Ishikawa H . Macrophage infiltration in the skin of patients with systemic sclerosis. J Rheumatol. (1992) 19:1202–6. 1404154

[26] Binai N O’Reilly S Griffiths B van Laar JM Hügle T . Differentiation potential of CD14+ monocytes into myofibroblasts in patients with systemic sclerosis. PloS One. (2012) 7:e33508. doi: 10.1371/journal.pone.0033508. PMID: 22432031 PMC3303833

[27] Sack U Stiehl P Geiler G . Distribution of macrophages in rheumatoid synovial membrane and its association with basic activity. Rheumatol Int. (1994) 13:181–6. doi: 10.1007/BF00390265. PMID: 8202661

[28] Tak PP Smeets TJ Daha MR Kluin PM Meijers KA Brand R . Analysis of the synovial cell infiltrate in early rheumatoid synovial tissue in relation to local disease activity. Arthritis Rheum. (1997) 40:217–25. doi: 10.1002/art.1780400206. PMID: 9041933

[29] Mulherin D Fitzgerald O Bresnihan B . Synovial tissue macrophage populations and articular damage in rheumatoid arthritis. Arthritis Rheum. (1996) 39:115–24. doi: 10.1002/art.1780390116. PMID: 8546720

[30] Janossy G Panayi G Duke O Bofill M Poulter LW Goldstein G . Rheumatoid arthritis: a disease of T-lymphocyte/macrophage immunoregulation. Lancet. (1981) 2:839–42. doi: 10.1016/s0140-6736(81)91107-7. PMID: 6116956

[31] Marée AFM Komba M Dyck C Łabecki M Finegood DT Edelstein-Keshet L . Quantifying macrophage defects in type 1 diabetes. J Theor Biol. (2005) 233:533–41. doi: 10.1016/j.jtbi.2004.10.030. PMID: 15748914

[32] Min D Brooks B Wong J Salomon R Bao W Harrisberg B . Alterations in monocyte CD16 in association with diabetes complications. Mediators Inflammation. (2012) 2012:649083. doi: 10.1155/2012/649083. PMID: 23316106 PMC3536440

[33] Katsiari CG Liossis S-N Souliotis VL Dimopoulos AM Manoussakis MN Sfikakis PP . Aberrant expression of the costimulatory molecule CD40 ligand on monocytes from patients with systemic lupus erythematosus. Clin Immunol. (2002) 103:54–62. doi: 10.1006/clim.2001.5172. PMID: 11987985

[34] Blanco P Palucka AK Gill M Pascual V Banchereau J . Induction of dendritic cell differentiation by IFN-alpha in systemic lupus erythematosus. Science. (2001) 294 (5546):1540–1543. doi: 10.1126/science.1064890. PMID: 11711679. 11711679

[35] Ma W-T Gao F Gu K Chen D-K . The role of monocytes and macrophages in autoimmune diseases: a comprehensive review. Front Immunol. (2019) 10:1140. doi: 10.3389/fimmu.2019.01140. PMID: 31178867 PMC6543461

[36] Zhou J Zhang S Guo C . Crosstalk between macrophages and natural killer cells in the tumor microenvironment. Int Immunopharmacol. (2021) 101:108374. doi: 10.1016/j.intimp.2021.108374. PMID: 34824036

[37] Miettinen M Matikainen S Vuopio-Varkila J Pirhonen J Varkila K Kurimoto M . Lactobacilli and streptococci induce interleukin-12 (IL-12), IL-18, and gamma interferon production in human peripheral blood mononuclear cells. Infect Immun. (1998) 66:6058–62. doi: 10.1128/IAI.66.12.6058-6062.1998. PMID: 9826398 PMC108774

[38] Hooda V Sharma A . Interactions of NK cells and macrophages: from infections to cancer therapeutics. Immunology. (2025) 174:287–95. doi: 10.1111/imm.13886. PMID: 39739619

[39] Krneta T Gillgrass A Poznanski S Chew M Lee AJ Kolb M . M2-polarized and tumor-associated macrophages alter NK cell phenotype and function in a contact-dependent manner. J Leukoc Biol. (2017) 101:285–95. doi: 10.1189/jlb.3A1215-552R. PMID: 27493241

[40] Vitale C Bottino C Castriconi R . Monocyte and macrophage in neuroblastoma: blocking their pro-tumoral functions and strengthening their crosstalk with natural killer cells. Cells. (2023) 12:885. doi: 10.3390/cells12060885. PMID: 36980226 PMC10047506

[41] Boyerinas B Jochems C Fantini M Heery CR Gulley JL Tsang KY . Antibody-dependent cellular cytotoxicity activity of a novel anti-PD-L1 antibody avelumab (MSB0010718C) on human tumor cells. Cancer Immunol Res. (2015) 3:1148–57. doi: 10.1158/2326-6066.CIR-15-0059. PMID: 26014098 PMC4739754

[42] Zhou Z Zhang C Zhang J Tian Z . Macrophages help NK cells to attack tumor cells by stimulatory NKG2D ligand but protect themselves from NK killing by inhibitory ligand Qa-1. PloS One. (2012) 7:e36928. doi: 10.1371/journal.pone.0036928. PMID: 22629344 PMC3356357

[43] Sun L Kees T Almeida AS Liu B He X-Y Ng D . Activating a collaborative innate-adaptive immune response to control metastasis. Cancer Cell. (2021) 39:1361–1374.e9. doi: 10.1016/j.ccell.2021.08.005. PMID: 34478639 PMC8981964

[44] Malhotra A Shanker A . NK cells: immune cross-talk and therapeutic implications. Immunotherapy. (2011) 3:1143–56. doi: 10.2217/imt.11.102. PMID: 21995569 PMC3230271

[45] Desimio MG Covino DA Rivalta B Cancrini C Doria M . The role of NK cells in EBV infection and related diseases: current understanding and hints for novel therapies. Cancers (Basel). (2023) 15:1914. doi: 10.3390/cancers15061914. PMID: 36980798 PMC10047181

[46] Gianchecchi E Delfino DV Fierabracci A . NK cells in autoimmune diseases: linking innate and adaptive immune responses. Autoimmun Rev. (2018) 17:142–54. doi: 10.1016/j.autrev.2017.11.018. PMID: 29180124

[47] Sohlberg E Malmberg K-J . The innate power of natural killer cells in cancer therapy. Nat Med. (2025) 31 (6):1755–1756. doi: 10.1038/s41591-025-03712-9. PMID: 40355615

[48] Lima MZT Bastos DA Mattedi RL Dzik C Jardim DLF Coelho R . Infiltrating natural killer cells influence the efficacy of BCG immunotherapy in non-muscle-invasive bladder cancer. Pathol Res Pract. (2025) 270:155997. doi: 10.1016/j.prp.2025.155997. PMID: 40349568

[49] Hu Z Zheng M Guo Z Zhou W Zhou W Yao N . Single-cell sequencing reveals distinct immune cell features in cutaneous lesions of pemphigus vulgaris and bullous pemphigoid. Clin Immunol. (2024) 263:110219. doi: 10.1016/j.clim.2024.110219. PMID: 38631594

[50] Cutolo M Soldano S Smith V Gotelli E Hysa E . Dynamic macrophage phenotypes in autoimmune and inflammatory rheumatic diseases. Nat Rev Rheumatol. (2025) 21:546–65. doi: 10.1038/s41584-025-01279-w. PMID: 40721670

[51] Strizova Z Benesova I Bartolini R Novysedlak R Cecrdlova E Foley LK . M1/M2 macrophages and their overlaps – myth or reality? Clin Sci (Lond). (2023) 137:1067–93. doi: 10.1042/CS20220531. PMID: 37530555 PMC10407193

[52] Funes SC Rios M Escobar-Vera J Kalergis AM . Implications of macrophage polarization in autoimmunity. Immunology. (2018) 154 (2):186–95. doi: 10.1111/imm.12910 PMC5980179 29455468

[53] Thiesen S Janciauskiene S Uronen-Hansson H Agace W Högerkorp C-M Spee P . CD14(hi)HLA-DR(dim) macrophages, with a resemblance to classical blood monocytes, dominate inflamed mucosa in Crohn’s disease. J Leukoc Biol. (2014) 95:531–41. doi: 10.1189/jlb.0113021. PMID: 24212097

[54] Wildenberg ME Welzen-Coppens JMC van Helden-Meeuwsen CG Bootsma H Vissink A van Rooijen N . Increased frequency of CD16+ monocytes and the presence of activated dendritic cells in salivary glands in primary Sjögren syndrome. Ann Rheum Dis. (2009) 68:420–6. doi: 10.1136/ard.2008.087874. PMID: 18397959

[55] Ciccia F Alessandro R Rodolico V Guggino G Raimondo S Guarnotta C . IL-34 is overexpressed in the inflamed salivary glands of patients with Sjogren’s syndrome and is associated with the local expansion of pro-inflammatory CD14(bright)CD16+ monocytes. Rheumatol (Oxford). (2013) 52:1009–17. doi: 10.1093/rheumatology/kes435. PMID: 23392590

[56] Bennett L Palucka AK Arce E Cantrell V Borvak J Banchereau J . Interferon and granulopoiesis signatures in systemic lupus erythematosus blood. J Exp Med. (2003) 197:711–23. doi: 10.1084/jem.20021553. PMID: 12642603 PMC2193846

[57] Yang Z Ming X-F . Functions of arginase isoforms in macrophage inflammatory responses: Impact on cardiovascular diseases and metabolic disorders. Front Immunol. (2014) 5:533. doi: 10.3389/fimmu.2014.00533. PMID: 25386179 PMC4209887

[58] Ahamada MM Jia Y Wu X . Macrophage polarization and plasticity in systemic lupus erythematosus. Front Immunol. (2021) 12:734008. doi: 10.3389/fimmu.2021.734008. PMID: 34987500 PMC8721097

[59] Higashi-Kuwata N Makino T Inoue Y Takeya M Ihn H . Alternatively activated macrophages (M2 macrophages) in the skin of patient with localized scleroderma. Exp Dermatol. (2009) 18:727–9. doi: 10.1111/j.1600-0625.2008.00828.x. PMID: 19320738

[60] Lu C-H Lai C-Y Yeh D-W Liu Y-L Su Y-W Hsu L-C . Involvement of M1 macrophage polarization in endosomal Toll-like receptors activated psoriatic inflammation. Mediators Inflammation. (2018) 2018:3523642. doi: 10.1155/2018/3523642. PMID: 30647534 PMC6311781

[61] Yuan Q Yang W Zhang X . Immune cells in pemphigus vulgaris and bullous pemphigoid: From pathogenic roles to targeting therapies. Int Immunopharmacol. (2023) 123:110694. doi: 10.1016/j.intimp.2023.110694. PMID: 37523970

[62] Sain N Hooda V Singh A Gupta S Arava S Sharma A . Macrophage inhibitory factor alters the functionality of macrophages and their involvement in disease pathogenesis of active generalized vitiligo patients. Cytokine. (2024) 176:156516. doi: 10.1016/j.cyto.2024.156516 38340551

[63] Li J Hsu H-C Mountz JD . The dynamic duo–inflammatory M1 macrophages and Th17 cells in rheumatic diseases. J Orthop Rheumatol. (2013) 1:4. doi: 10.13188/2334-2846.1000002. PMID: 25309946 PMC4193941

[64] Harbour SN DiToro DF Witte SJ Zindl CL Gao M Schoeb TR . Th17 cells require ongoing classic IL-6 receptor signaling to retain transcriptional and functional identity. Sci Immunol. (2020) 5:eaaw2262. doi: 10.1126/sciimmunol.aaw2262. PMID: 32680955 PMC7843024

[65] Kimura A Naka T Kishimoto T . IL-6-dependent and -independent pathways in the development of interleukin 17-producing T helper cells. Proc Natl Acad Sci. (2007) 104:12099–104. doi: 10.1073/pnas.0705268104. PMID: 17623780 PMC1924582

[66] Pyrillou K Burzynski LC Clarke MCH . Alternative pathways of IL-1 activation, and its role in health and disease. Front Immunol. (2020) 11:613170. doi: 10.3389/fimmu.2020.613170. PMID: 33391283 PMC7775495

[67] Vorwerk G Zahn S Bieber T Wenzel J . NKG2D and its ligands as cytotoxic factors in cutaneous lupus erythematosus. Exp Dermatol. (2021) 30:847–52. doi: 10.1111/exd.14311. PMID: 33687107

[68] Nedvetzki S Sowinski S Eagle RA Harris J Vély F Pende D . Reciprocal regulation of human natural killer cells and macrophages associated with distinct immune synapses. Blood. (2007) 109:3776–85. doi: 10.1182/blood-2006-10-052977. PMID: 17218381

[69] Michel T Hentges F Zimmer J . Consequences of the crosstalk between monocytes/macrophages and natural killer cells. Front Immunol. (2013) 3:403. doi: 10.3389/fimmu.2012.00403 23316194 PMC3539656

[70] Bellora F Castriconi R Dondero A Reggiardo G Moretta L Mantovani A . The interaction of human natural killer cells with either unpolarized or polarized macrophages results in different functional outcomes. Proc Natl Acad Sci. (2010) 107:21659–64. doi: 10.1073/pnas.1007654108. PMID: 21118979 PMC3003022

