# Supplementary material for: NK cell hyperactivation drives macrophage repolarization and limits M2 bias in pemphigus vulgaris
Source: Front Immunol. 2026 May 8;17:1774059. doi: 10.3389/fimmu.2026.1774059 (PMC13194483; doi:10.3389/fimmu.2026.1774059)
Supplement: Supplementary file 1 [file Supplementaryfile1.docx]

eTable 1: List of primer sequences used for mRNA expression analysis in real time PCR

| *TGFB1* | Forward | CCAGCGAGCCAGGCAG | 165 |
| --- | --- | --- | --- |
|  | Reverse | ATTTGAGATGTATGTGTCTTCTTCG |  |
| *TNF* | Forward | CAAACTGGTGCTCAAGGCCC | 198 |
|  | Reverse | GCGTCCCAAAGTAGGAGAGG |  |
| *IL10* | Forward | GCTGGAGGACTTTAAGGGTTAC | 106 |
|  | Reverse | GATGTCTGGGTCTTGGTTCTC |  |
| *IL23A* | Forward | GCAGATTCCAAGCCTCAGTC | 152 |
|  | Reverse | CCTTGAGCTGCTGCCCTTTAG |  |
| *IL 1B* | Forward | CGGCGCTGTCATCGATTTCTT | 168 |
|  | Reverse | AGTCGCCACCCTGATGTCTC |  |
| *MICA* | Forward | TGCCTGATGGGAATGGAACC | 184 |
|  | Reverse | CCAGCAGCAACAGCAGAAAC |  |
| *CD86* | Forward | GGACTTTAAGGGTTACCTGGGTT | 129 |
|  | Reverse | CGGCCTTGCTCTTGTTTTCA |  |
| *CD206* | Forward | GCCTCGTTGTTTTGCGTCTT | 123 |
|  | Reverse | GAGAACAGCACCCGGAATGA |  |
| *NOS2* | Forward | TGAGCCCCTTCATCAATGCTT | 174 |
|  | Reverse | AGGCTCATGGTGCATACAGT |  |
| *ARG1* | Forward | ACAGTGAAATCAATCATCCCCA | 224 |
|  | Reverse | ATCTTCCACCTCCCAAAAGCTA |  |
| *18S* | Forward | GTAACCCGTTGAACCCCATT | 151 |
|  | Reverse | CCATCCAATCGGTAGTAGCG |  |

eTable 2: Demographic details of the study subjects

| Category | Total |
| --- | --- |
| PV Patients | |
| Number | 35 |
| Median Age | 44 years  (23 to 65 years) |
| Sex | 19F/ 16M |
| PDAI Severity Score |  |
| Moderate (<15) | 12 |
| Significant (15-45) | 15 |
| Extensive (>45) | 8 |
| Healthy controls | |
| Number  *Age and sex matched | 35  (19F/16M) |
| Median Age | 42years |

*M= Male, F= Female, PV= Pemphigus vulgaris, PDAI= Pemphigus Disease Area Index*

**PDAI Scoring**: The patients’ severity level was calculated using PDAI score. Out of total 250, 120 scoring is for skin activity, 10 scoring for scalp activity and remaining 120 is score for mucosal activity. Total scoring determines the patient’s severity, where patients with score 0-15 are considered as moderate, 15-45 as significant and more than 45 comes under extensive severity.

**eTable 3:** **List of antibodies used in Flow cytometry**

| Marker | Fluorochrome | Company | Catalogue no. |
| --- | --- | --- | --- |
| CD14 | Super Bright 780 | Invitrogen | 78-0149-42 |
| CD11b | Brilliant Violet 421 | Invitrogen | 62-0113-42 |
| HLA-DR | APC-eflour 780 | Invitrogen | 47-9956-42 |
| CD64 | FITC | Invitrogen | CD6401 |
| CD86 | Super Bright 645 | Invitrogen | 64-0869-42 |
| CD163 | AlexaFlour700 | Invitrogen | 56-1639-42 |
| CD206 | PECy7 | Invitrogen | 25-2069-42 |
| NKG2D | Super Bright 436 (Brilliant Violet 421) | Invitrogen | 62587842 |
| KIR2D | PerCPCy5.5 | Invitrogen | 45158942 |
| MICA/B | PE | Invitrogen | 12-5788-42 |
| CD69 | PE Cy7 | Invitrogen | 25-0699-42 |
| Live/Dead^TM^ fixable blue dead cell stain | BUV496 | Invitrogen | L34962 |

**
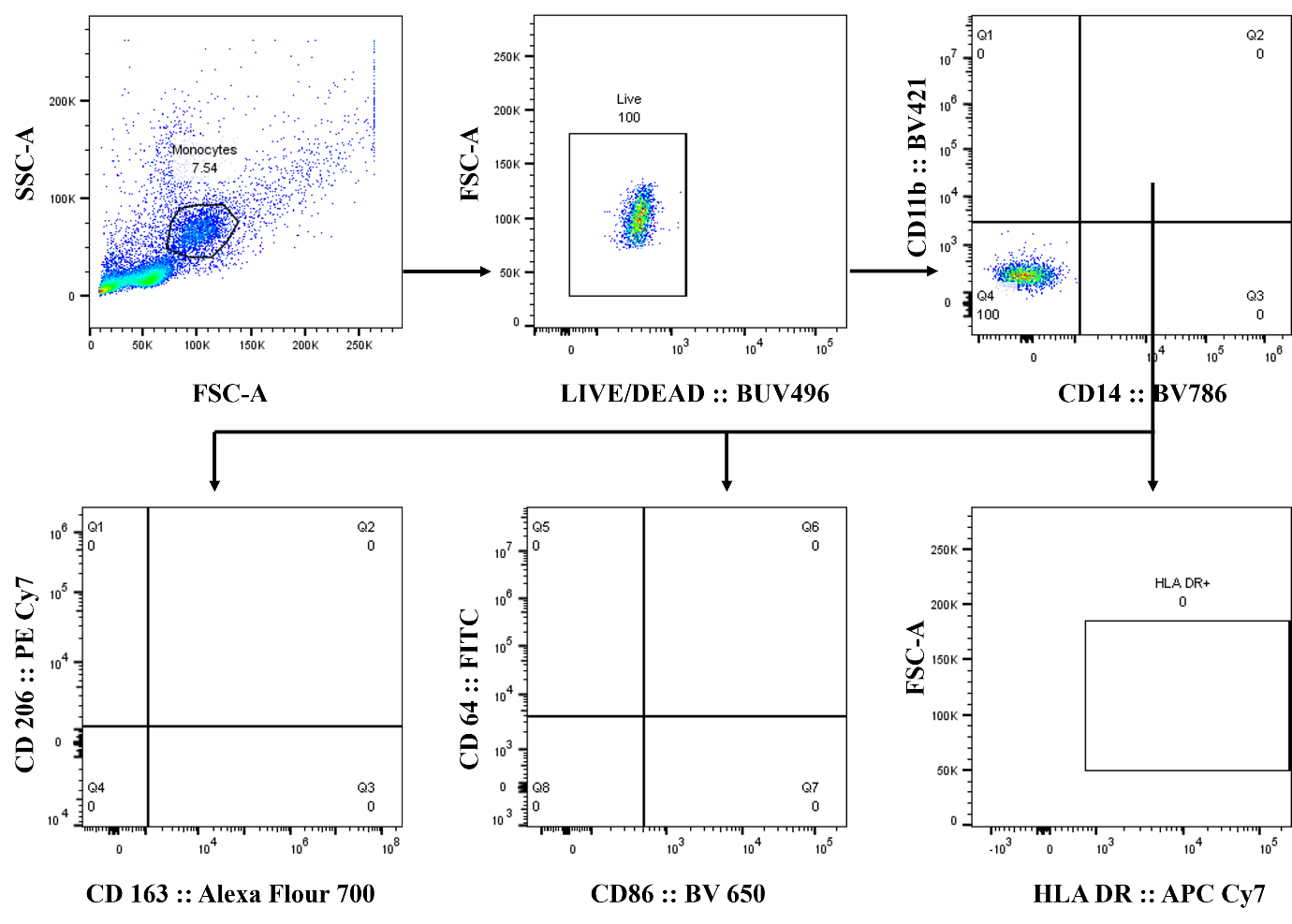
**

**eFigure 1**: **Gating strategy of macrophage like monocytes**

Gating strategy followed in flow cytometry to identify macrophage like monocytes in PBMCs. The dot plots are represented in pseudo color format. Monocytes were gated initially followed by selection of live population. This population was gated for dual positive population CD11b+ CD14+ cells known as macrophage like monocytes. This population was used to detect activation using HLA-DR marker and to categorize M1 and M2 macrophages related markers i.e. CD64 and CD86; CD163 and CD206 respectively. CD14 was tagged with BV 786, CD11b with BV 421, HLA DR with APC Cy7 and CD 64, CD86, CD163 and CD206 were tagged with Alexa flour700, PE Cy7, PE Texas red and BV 650 respectively.

**
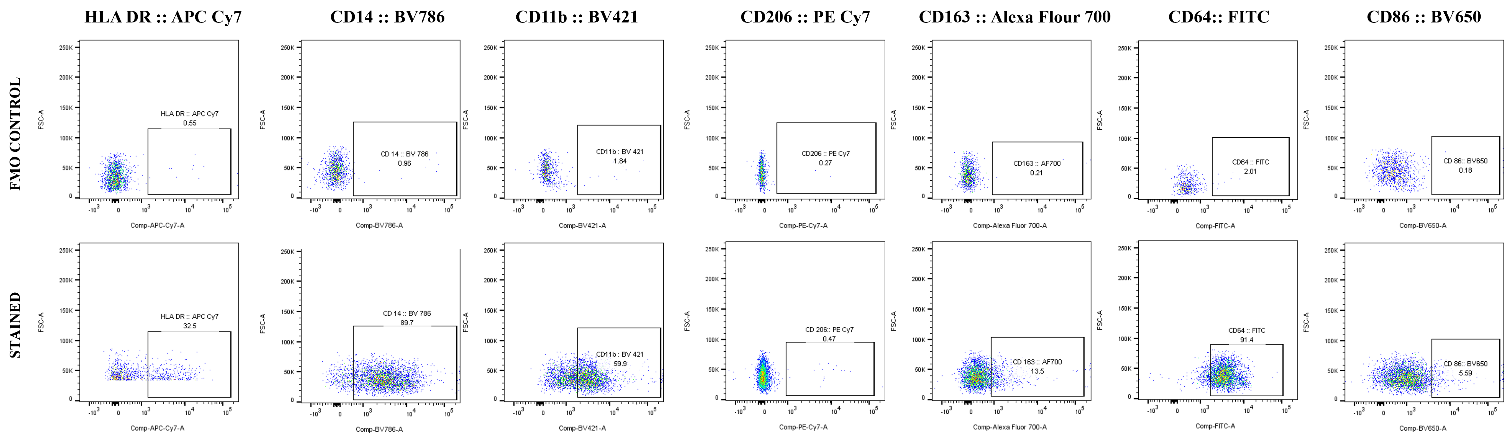
**

**eFigure 2: FMO controls for macrophage like monocytes**

The upper panel represents FMO control for the above-mentioned markers. The lower panel represents the stained sample of the respective FMO control.

**
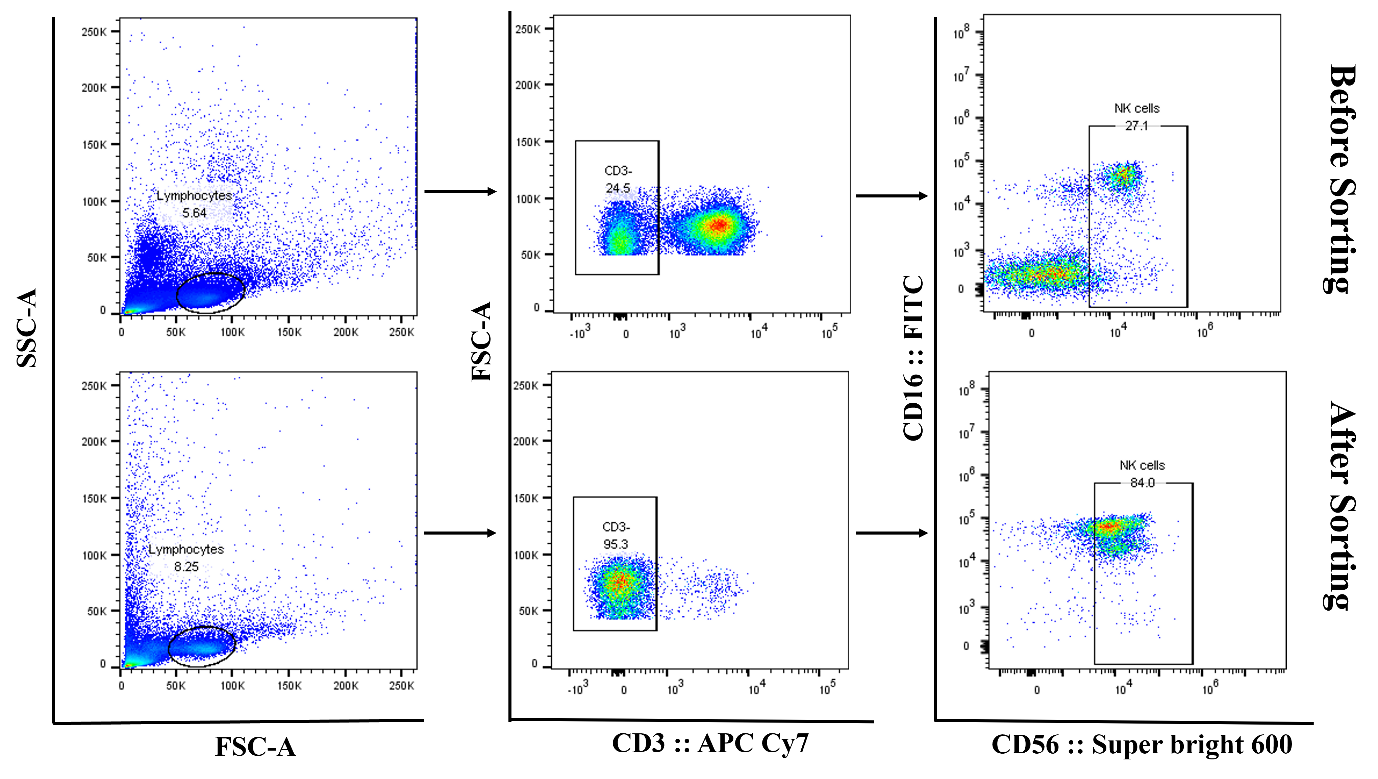
eFigure 3: Purity check for NK cells after MACS sorting**

PBMCs were used to isolate NK cells via negative selection MACS kit. Lymphocytes were gated followed by negative selection for CD3. CD3- cells were gated for CD56+ CD16+ NK cells. The flow cytometry dot plots are represented in pseudo colour format. The purity obtained was around 84% after sorting.


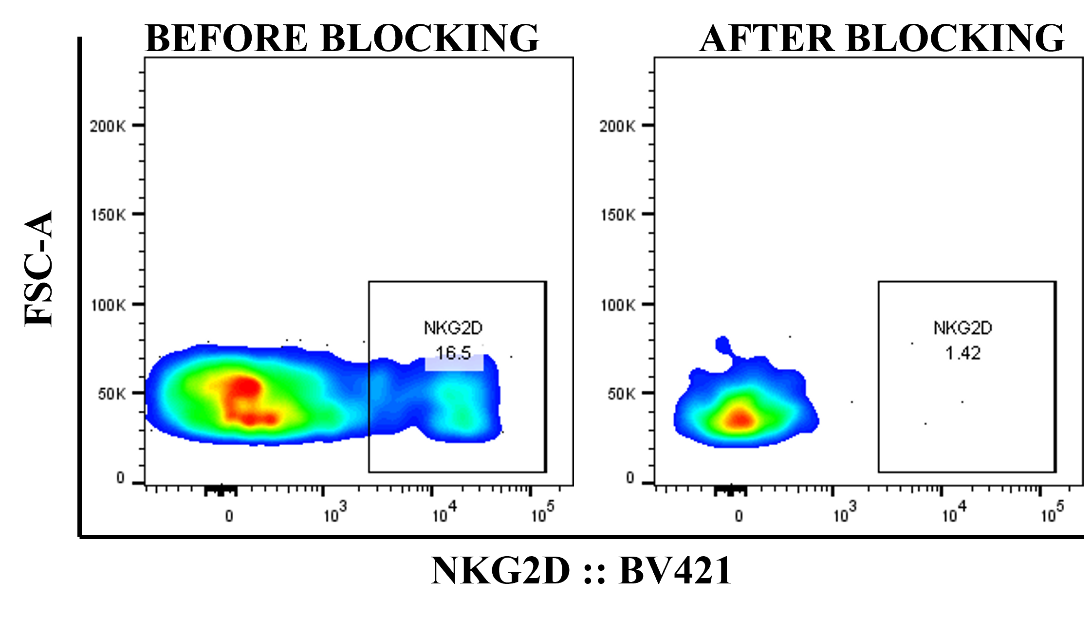


**eFigure 4: NKG2D Blocking on cultured NK cells**

The flow cytometry pseudo colour plot represents expression of NKG2D on cultured NK cells before and after NKG2D blockade.


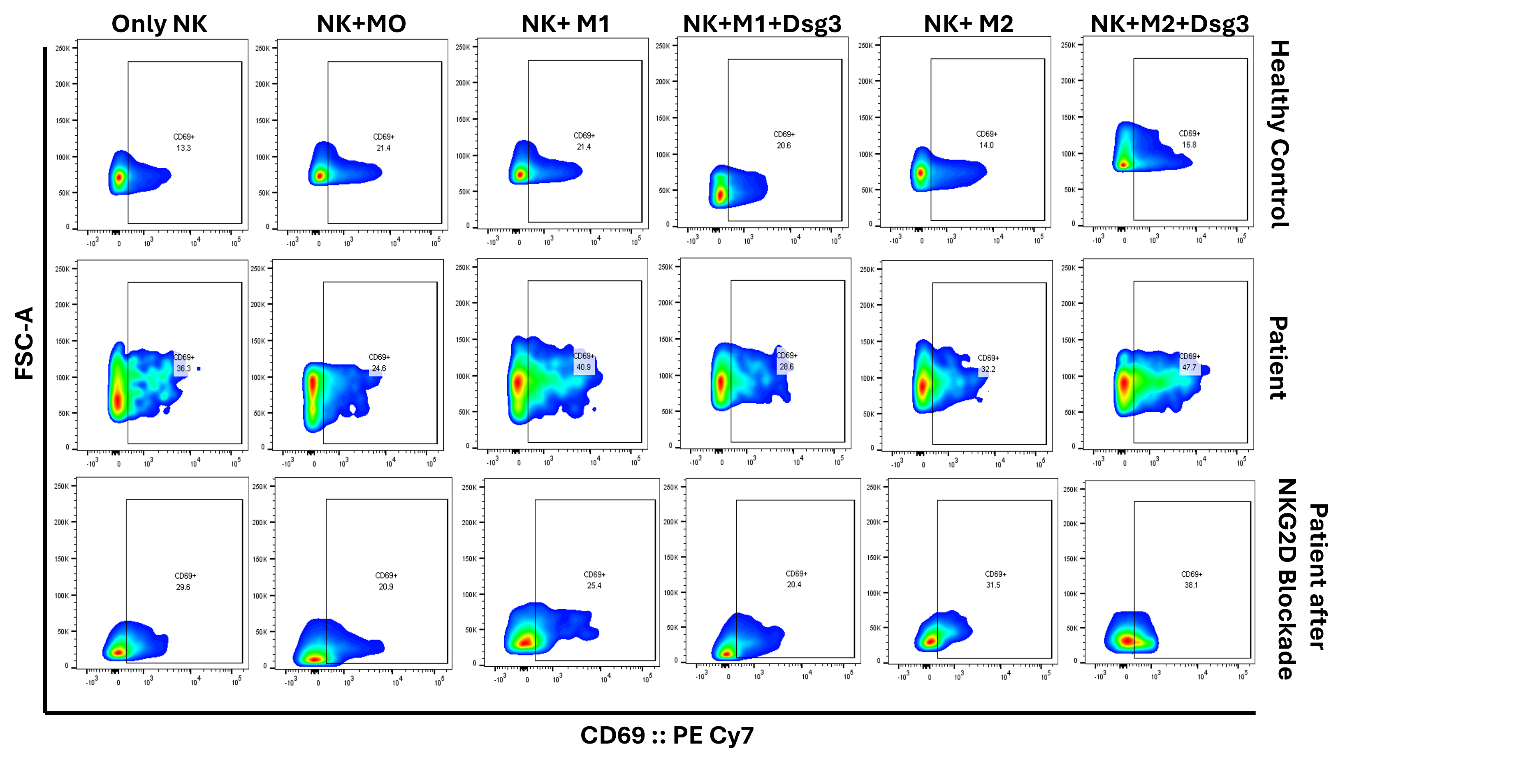


**eFigure 5: Representative flow cytometry plots of CD69- activation marker on cultured NK cells**

**
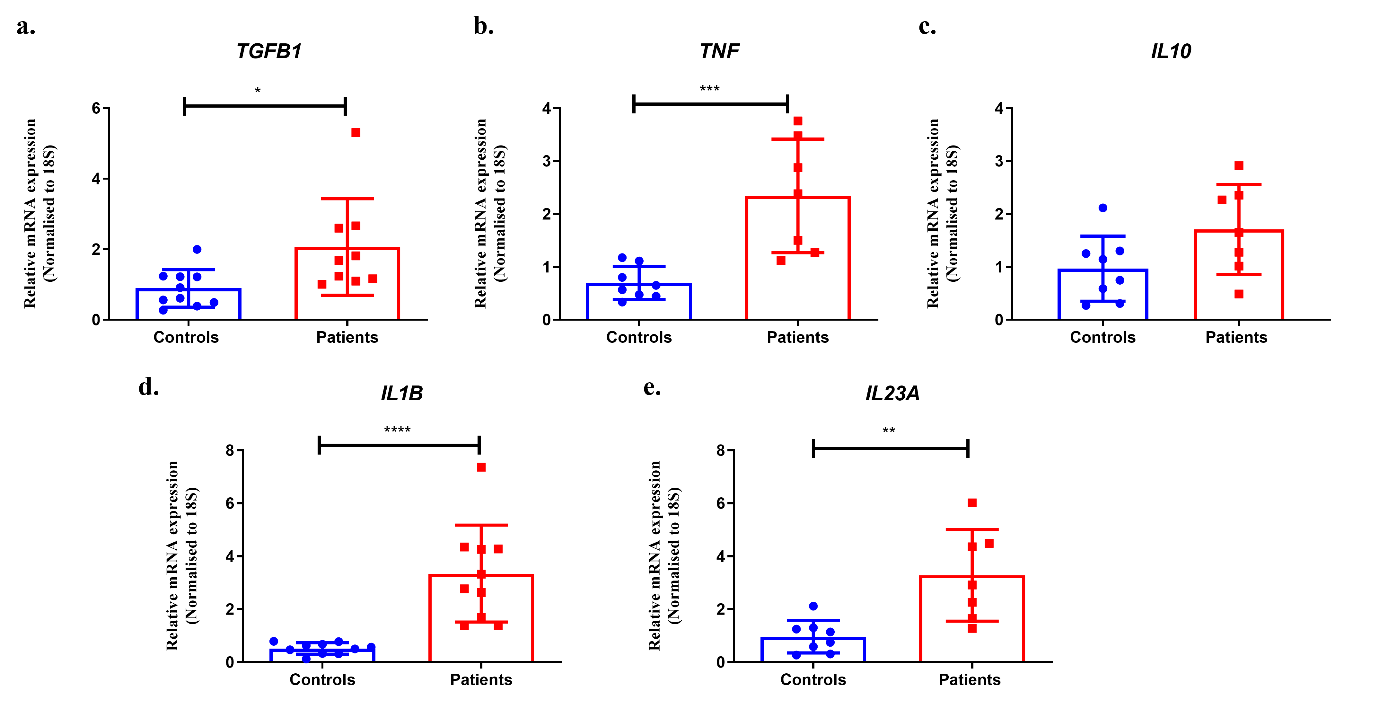
**

eFigure 6: Expression profile of macrophage associated markers in tissues.

mRNA expression of anti-inflammatory marker a.) TGFβ and c.), pro-inflammatory macrophage associated markers b.), d.) e.) in PV compared to HC. Bar graph with mean with SEM representation. ***p<0.001, ****p<0.0001, **p<0.005, *p<0.05, n= 10.


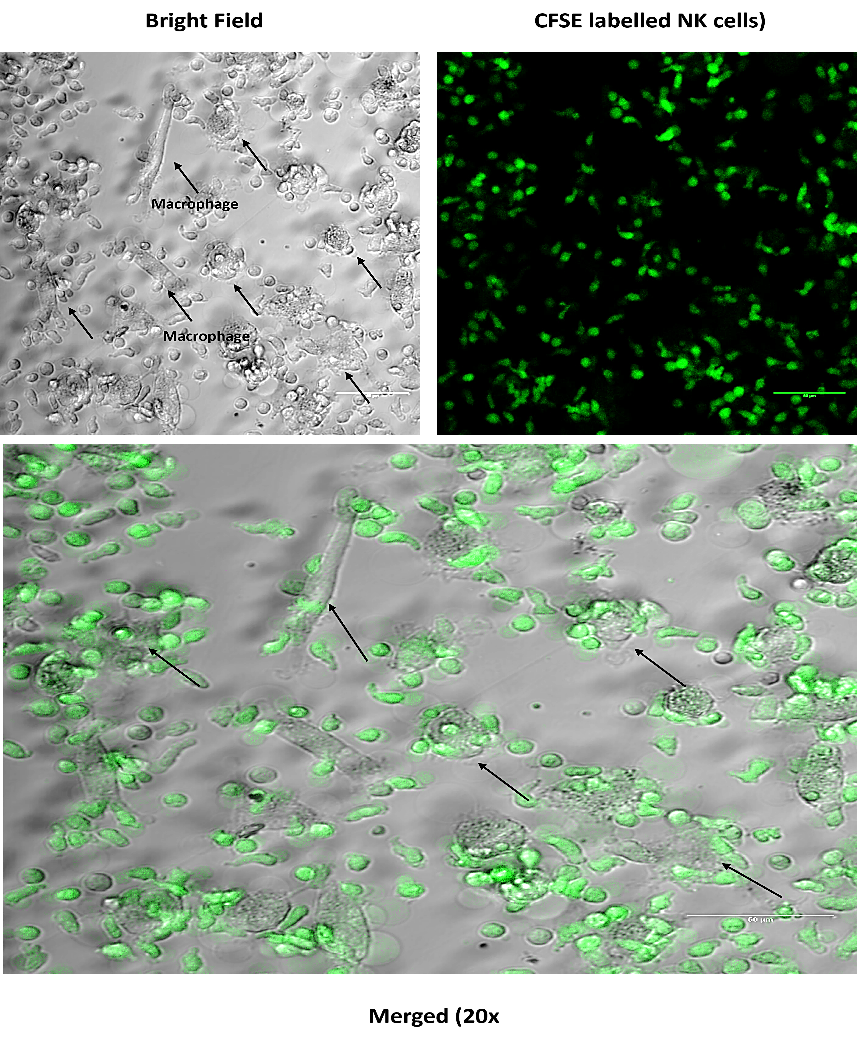


eFigure 7: Representation of NK cells and Macrophages interactions *in vitro*.

NK cells were labelled with CFSE to distinguish them from macrophages. Images were taken at 20x magnification, scale bar=50µm.

**
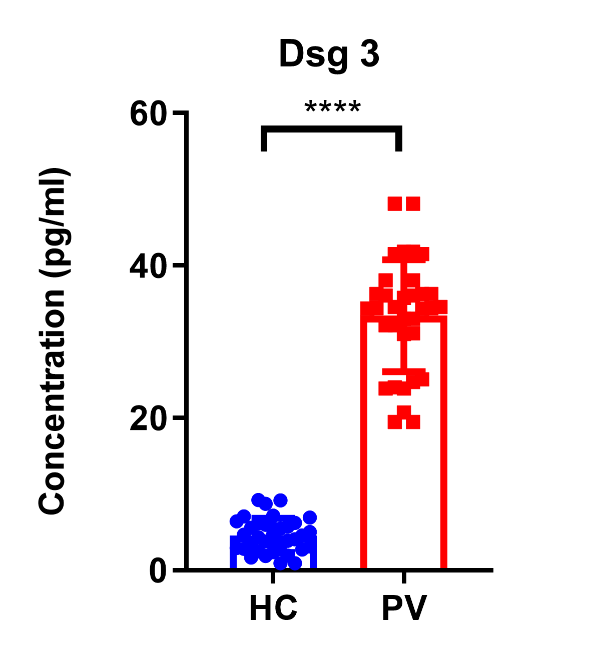
**

eFigure 8: Circulatory levels of anti-Dsg3 antibodies in the serum.

Column bar graph with mean with SD (standard deviation) representing anti-Dsg3 antibody levels which were significantly enhanced in PV compared to HC (healthy controls). **** p<0.0001, n=35.

**
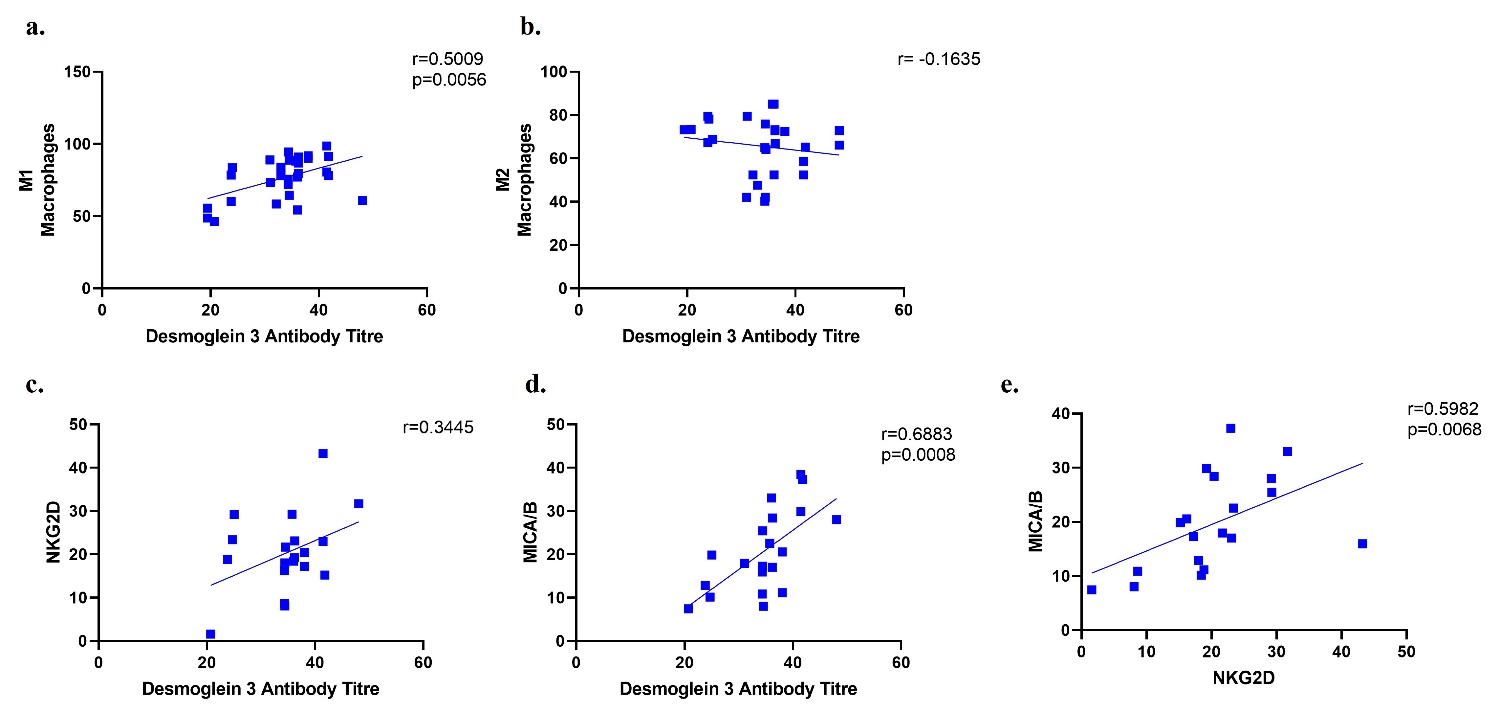
**

**eFigure 9: Correlation analysis of macrophage markers with Desmoglein 3**

a.) and b.) The scatter plot depicting correlation between M1 macrophages and M2 macrophages peripheral frequency of PV patients with desmoglein 3 antibody titres, respectively; c.) correlation between NKG2D on NK cells with desmoglein 3 antibody titres; d.) correlation between MICA/B on macrophages with desmoglein 3 antibody titres e. correlation between NKG2D and MICA/B. Correlation was performed using spearman’s correlation analysis. Line shows best fit regression. The strength of correlation is shown by r and significance by p values in figure.
